# Integrated transcriptomics and physio-biochemical analysis revealed key genes affecting the seed germination of *Leymus chinensis*

**DOI:** 10.3389/fpls.2025.1696194

**Published:** 2025-10-31

**Authors:** Yumei Feng, Wenlong Gong, Lemeng Liu, Chunyu Tian, Yanting Yang, Zinian Wu

**Affiliations:** ^1^ Institute of Grassland Research, Chinese Academy of Agricultural Sciences, Hohhot, China; ^2^ Key Laboratory of Forage Resources and Utilization, Ministry of Agriculture and Rural Affairs, Hohhot, China

**Keywords:** *Leymus chinensis*, seed germination, seed hulls, RNA-seq, transcription factor

## Abstract

*Leymus chinensis*, also known as alkali grass, is a perennial rhizomatous herbaceous plant with a significant ecological and economic value. However, its industrial development is hindered by low seed germination rates. Therefore, how to break the seed dormancy and improve the germination rate of *L. chinensis* seeds has become a hot topic nowadays. In this study, the increase of gibberellin (GA) content is one of the main factors promoting the seed germination of *L. chinensis*. Furthermore, to further explore the molecular mechanisms underlying seed germination regulation, RNA sequencing (RNA-seq) analysis of seeds at different developmental stages was carried out to explore the potential regulatory factors of seed hulls affecting the seed germination of *L. chinensis*. A total of 21,564, 21,010, and 31,795 differentially expressed genes (DEGs) were identified in “H_CK_0 *vs*. DH_CK_0”, “H_CK_2 *vs*. DH_CK_2”, and “H_CK_7 *vs*. DH_CK_7”, respectively. Kyoto Encyclopedia of Genes and Genomes (KEGG) analysis showed that the DEGs were significantly enriched in plant hormone signal transduction, starch and sucrose metabolism, and mitogen-activated protein kinase (MAPK) signaling pathways. Within these pathways, we identified several candidate genes potentially involved in seed hull-mediated germination regulation, including those encoding α-amylase (AMY1), β-amylase (BAM1), α-xylosidase (XYL1), β-glucosidase (BGLU10/45), auxin-responsive protein (IAA18), and transcription factors (WRKY, AP2/ERF, ABI2, bZIP, and NAC). These results provide valuable genetic resources for studying the molecular mechanism of *L. chinensis* seed germination.

## 1 Introduction

Seed germination is the process that begins with seed imbibition and ends with the radicle breaking through the seed hulls. During this process, seeds undergo a series of physiological and morphological changes (Penfield, 2017). In addition, seed germination is a complex biological process that is influenced by seed hulls, endogenous substances, and environmental signals, such as enzymes, endogenous hormones, light, temperature, and moisture (De Wit et al., 2016; Shu et al., 2016). Among these factors, the seed hulls play a critical role in regulating germination by acting as a physical and biochemical barrier. In an investigation into seed dormancy mechanisms in *Leymus chinensis*, it was identified that the seed hulls contributed to 28.4% of the factors inducing dormancy (He et al., 2016). The seed hulls not only protect the embryo from mechanical damage, pathogens, and extreme environmental conditions but also control the exchange of water, gases, and solutes necessary for germination (Nonogaki et al., 2010). In many plant species, seed hulls can impose dormancy, delaying germination until conditions are favorable for seedling survival (Nonogaki et al., 2010; Graeber et al., 2012). Understanding the mechanisms by which the seed coat influences germination is therefore essential for improving seed quality, enhancing germination rates, and optimizing plant establishment in both natural and agricultural systems.

Generally, seed hulls can affect the content of endogenous gibberellin (GA) and abscisic acid (ABA), thereby inhibiting seed germination (Kucera et al., 2005). ABA is a crucial plant hormone that plays a significant role in inhibiting seed germination, particularly under unfavorable environmental conditions (Nambara and Marion-Poll, 2005; Holdsworth et al., 2008; Finkelstein et al., 2008). The mechanism by which ABA inhibits seed germination involves several molecular and physiological processes. ABA accumulates in the seed tissues, particularly in the embryo and endosperm, where it binds to specific receptors. This binding triggers a signaling cascade that leads to the activation of various genes and proteins responsible for maintaining dormancy (Cutler et al., 2010). One of the key proteins activated by ABA is ABI5 (ABA-Insensitive 5), a transcription factor (TF) that regulates the expression of genes involved in seed dormancy (Lopez-Molina et al., 2002). It is well known that GA functions antagonistically to ABA, inhibits seed dormancy, and promotes seed germination (Graeber et al., 2012). GA, a hormone widely present in plants, is of great significance to various plant life activities (Forghani et al., 2018). Currently, it is widely recognized that GA can relieve the physiological dormancy of seeds and improve their germination rates (Finkelstein et al., 2008). It was found that GA mainly affected the content of H_2_O_2_ through changes in antioxidant-related enzymes and could lead to alterations in the coding genes of related antioxidant enzymes and H_2_O_2_ signaling, thus relieving dormancy (Zhuang et al., 2015). In addition, GA mainly relieves potato tuber dormancy by inducing the activity of α- and β-amylases, which play an important role in breaking seed dormancy (Rentzsch et al., 2012).

*L. chinensis*, known as alkali grass, is a perennial rhizome grass and one of the dominant species in meadow steppe. Exhibiting cold resistance, drought tolerance, salt tolerance, and good palatability, *L. chinensis* also functions as a medicinal plant with heat-clearing, detoxifying, swelling-reducing, and pain-relieving properties, thereby possessing high ecological and economic value. *L. chinensis* mainly reproduces asexually in natural habitats, supplemented by sexual reproduction (Wang et al., 2003; Chen and Wang, 2009). The speed of sexual reproduction is slow, and there are some phenomena, such as low heading rate, low seed setting rate, and low germination rate, which greatly reduce the yield of *L. chinensis* seeds (Guo et al., 2020; Liu et al., 2004). However, in recent years, with the development of animal husbandry, the demand for and quality of *L. chinensis* seeds have also increased (Ao et al., 2025). Therefore, how to break seed dormancy and improve the germination rate of *L. chinensis* seeds has become a hot topic. The removal of seed hulls to improve the germination rate of *L. chinensis* seeds has been reported, whereas few studies have focused on the mechanism of seed hulls regulating the germination rate of *L. chinensis* seeds at the transcriptional level.

In order to further identify the candidate genes of seed hulls regulating *L. chinensis* seed germination, we selected six groups, namely, DH_CK_0, DH_CK_2, DH_CK_7, H_CK_0, H_CK_2, and H_CK_7, for further transcriptome analysis. RNA sequencing (RNA-seq) analysis and weighted gene co-expression network analysis (WGCNA) were used to finally select the key genes related to seed hull regulation of seed germination. This study provides new insights into the potential genes of seed hulls regulating seed germination and helps to improve the seed germination rate in *L. chinensis* breeding.

## 2 Materials and methods

### 2.1 Plant materials and seed treatments

The mature seeds of *L. chinensis* were preserved by the Grassland Research Institute, Chinese Academy of Agricultural Sciences. All *L. chinensis* seeds were sequentially disinfected in 5% sodium hypochlorite and 75% alcohol for 3 min, followed by rinsing four times with sterile water. The seeds were placed in a sterilized Petri dish with three layers of filter paper for subsequent use. The de-hulled (DH-CK) and hulled (H-CK) seeds (50 seeds per sample) were soaked in sterile water for 12 h, respectively. All the treatments contained three biological replicates.

### 2.2 Seed germination assays

The de-hulled and hulled seeds (50 seeds per sample) were evenly placed in sterile Petri dishes, respectively. The Petri dishes were placed in an incubator at 25°C, 16 h light/8 h dark, and continuously cultured for 21 days. The number of germinations was counted every day. The germination potential (GP) was the number of germinated seeds on the 6th day divided by the total number of tested seeds. The first 7 days and total germination rate (GR) were calculated. The germination index (GI) was calculated (Equation 1). *G_t_* refers to the number of germinations on day *t* (21 days) of time, *D_t_* refers to the corresponding number of germination days, and ∑ refers to the sum. All the experiments contained three biological replicates.


(1) $$Germination\;index\;\left(GI\right)=\sum G_{t}/D_{t}$$

### 2.3 Samples for physiological indexes determination and transcriptome

A total of 300 de-hulled and hulled seeds were soaked in sterile water for 12 h, respectively. After treatment, the Petri dish containing seeds was placed in the incubator for normal germination. Subsequently, samples were collected at 0, 2, and 7 days after germination, respectively. These samples were defined as DH_CK_0, DH_CK_2, DH_CK_7, H_CK_0, H_CK_2, and H_CK_7, respectively. Physiological indexes, including IAA, ABA, GA3, soluble protein, soluble sugar contents, amylase, lipase, and neutral protease activity, were measured by Suzhou Comin Biotech Co., Ltd. (Suzhou, China). Transcriptomic analyses were performed by Shanghai OE Biotech Co., Ltd. (Shanghai, China). All of the experiments contained three replicates.

### 2.4 Transcriptome sequencing and analysis

Transcriptome sequencing of 18 samples was carried out using an Illumina HiSeq platform. To obtain high-quality reads for subsequent analysis, further quality filtering of the raw data is required. Firstly, Trimmomatic software was used for quality control and removal of adaptors. On this basis, low-quality bases and *N* bases were filtered out, and finally, high-quality clean reads were obtained. The paired-end splicing method of Trinity (version 2.4) software was used for *de novo* assembly to obtain transcript sequences. According to the sequence similarity and length, the longest one was selected as a unigene. DIAMOND software was used to compare unigenes to NR, KOG, Gene Ontology (GO), Swiss-Prot, eggNOG, and Kyoto Encyclopedia of Genes and Genomes (KEGG) database. The HMMER software was used to compare the Pfam database for functional analysis of unigene. FPKM and count of unigene were analyzed by bowtie 2 and express software. The estimate Size Factors function of the DESeq R package was used to standardize the data, and the nbinomTest function was used to calculate the *p*-value and fold change values of the difference comparison. Differentially expressed unigenes were selected with *p*<0.05 and a fold change greater than 2. Then, GO and KEGG enrichment analysis of differentially expressed unigenes was performed to determine the biological functions or pathways mainly affected by these unigenes. The raw data of RNA-seq were uploaded to the NCBI GEO database (accession number GSE308005).

### 2.5 WGCNA

A total of 18 samples and nine phenotypes were used to construct a phenotype weighted co-expression network. A total of 4,682 differentially expressed genes (DEGs) were submitted to WGCNA with standard deviation ≤ 0.3. The Pearson correlation algorithm was used to calculate the correlation coefficient and *p*-value between module characteristic genes and traits to screen the modules related to each trait (|correlation coefficient| ≥ 0.3, *p*<0.05). For module core genes analysis, the top 50 genes with the highest connectivity in each module were selected to show the relationship between these genes.

### 2.6 RNA extraction and qRT-PCR

Total RNA was extracted using Trizol reagent (Invitrogen, CA, USA) according to the manufacturer’s protocol. The cDNA was synthesized by the FastKing RT Kit (with gDNase) and the FastKing cDNA Kit [Tiangen Biotech (Beijing) Co., Ltd.]. The total reaction mixture for qRT-PCR was 20 μL, including 1 μL of 50 ng/μL cDNA, 10 μL of SYBR (TaKaRa), 0.4 μL of 10 µM primer F/primer R, and 8.2 μL of RNase-free water. The actin gene was used as the internal control for normalization. The relative expression was analyzed by the 2^−ΔΔCT^ method (Livak and Schmittgen, 2001). All data were obtained from three independent biological replicates. The sequences of primers are listed in **Supplementary Table S1**.

### 2.7 Statistical analysis

The GraphPad Prism 5 (GraphPad Software Inc., San Diego, CA, USA) was used to analyze the statistical significance (*t*-test). *p*<0.05, 0.01, or 0.001 was regarded as significantly different from the control. All data were obtained from three replicates.

## 3 Results

### 3.1 Effect of seed hulls on seed germination

**Figure 1:**
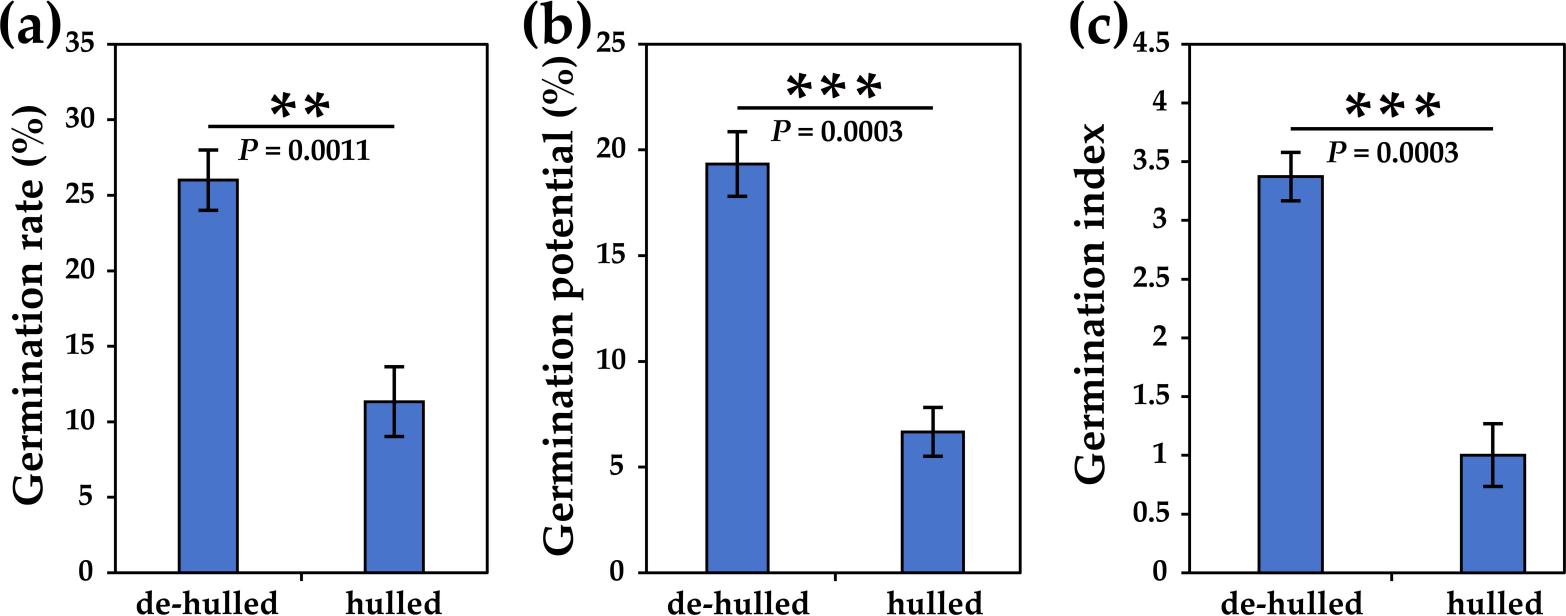
The effects of seed hulls on the seed germination of *L. chinensis*. **(a)** Total germination rate at 21 days. **(b)** Germination potential. **(c)** Germination index. The data were retrieved from three biological replicates. Data are the mean ± SD of three biological repeats. **p<0.01, ***p<0.001 (*t*-tests).

In order to explore the effect of seed hulls on the germination of *L. chinensis* seeds, we counted the germination rate, germination potential, and germination index, respectively. The results showed that the germination rate of hulled seeds was significantly lower than that of de-hulled seeds (approximately 44%, *p*=0.0011) (**Figure 1a**). In addition, seed germination potential showed a similar trend compared to the germination rate (approximately 35%, *p*=0.0003) (**Figure 1b**). Furthermore, the germination index of de-hulled seeds was significantly higher than that of hulled seeds (approximately 3.36-fold, *p*=0.0003), indicating that the uniformity of seed germination was good (**Figure 1c**).

### 3.2 RNA sequencing analysis

**Figure 2:**
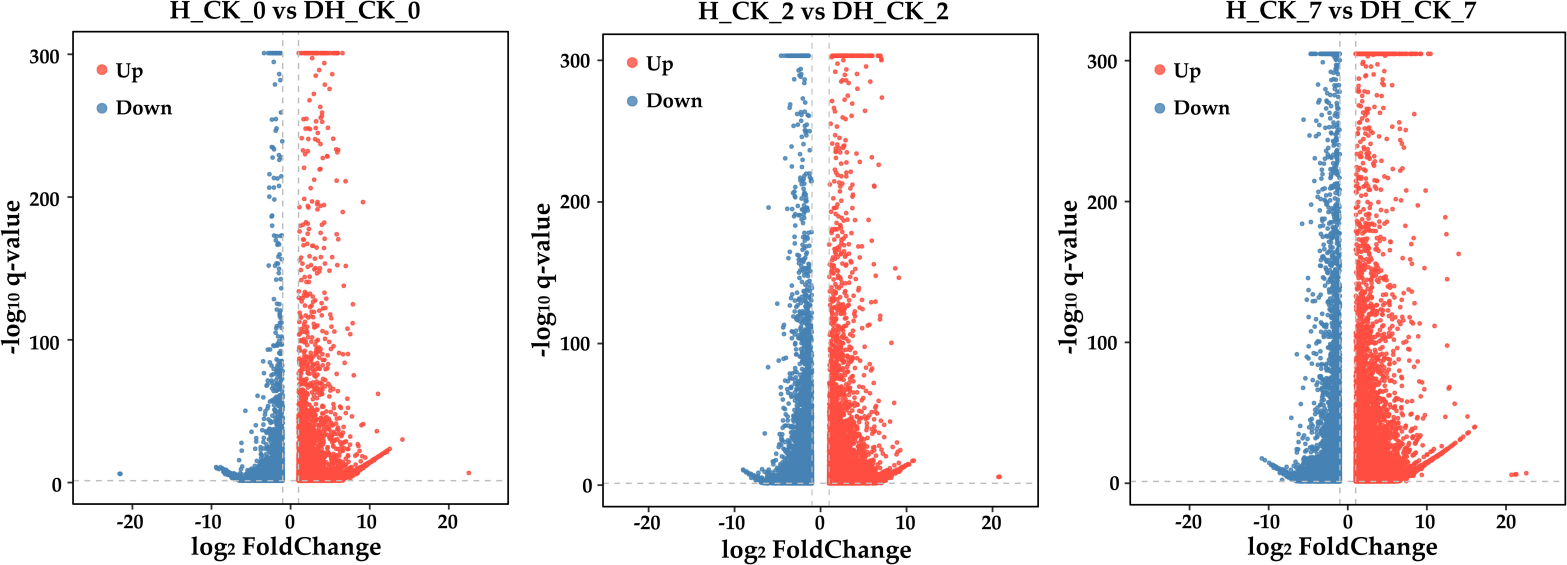
Volcano plots of DEGs’ upregulation and downregulation. DH_CK_0, DH_CK_2, and DH_CK_7 represented the de-hulled seeds treated with distilled water germinated for 0, 2, and 7 days, respectively. H_CK_0, H_CK_2, and H_CK_7 represented the hulled seeds treated with distilled water germinated for 0, 2, and 7 days, respectively.

**Figure 3:**
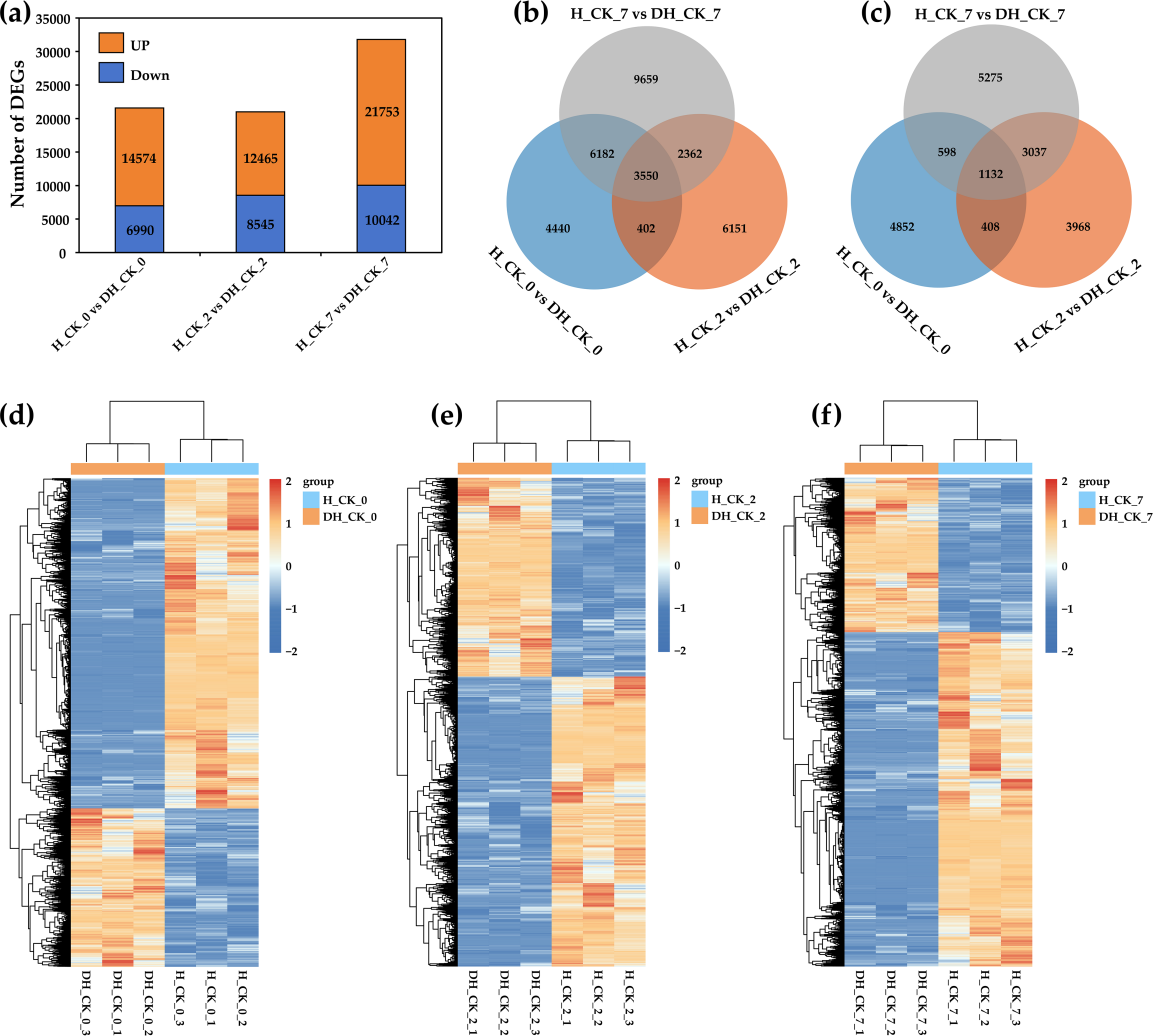
Characteristics of DEGs related to seed germination. **(a)** The bar diagram represented the number of DEGs in the three comparison groups. **(b)** Venn diagram represented the overlap of DEGs’ upregulation in the three comparison groups. **(c)** Venn diagram represented the overlap of DEGs’ downregulation in the three comparison groups. **(d–f)** The expression patterns of DEGs in each comparison group. DH_CK_0, DH_CK_2, and DH_CK_7 represented the de-hulled seeds treated with distilled water germinated for 0, 2, and 7 days, respectively. H_CK_0, H_CK_2, and H_CK_7 represented the hulled seeds treated with distilled water germinated for 0, 2, and 7 days, respectively.

In order to explore how seed hulls affect the germination of *L. chinensis* seeds, 18 samples were used for RNA-seq analysis. A total of 216.39 Gb clean data were generated with Q30 values ranging from 94.47% to 97.29%, and the mean GC content was 56.21% (**Supplementary Table S2**). Principal component analysis (PCA) indicated that PC1 and PC2 were able to explain 50.21% and 28.06% of the variation, respectively (**Supplementary Figure S1a**). In addition, the Pearson correlation analysis of RNA-seq data showed that the repeatability of the data is very good (**Supplementary Figure S1b**). These results suggested a high level of confidence in the RNA-seq data. In order to determine the changes of RNA-seq data during the germination of de-hulled and hulled seeds, we analyzed the DEGs in DH_CK_0, DH_CK_2, DH_CK_7, H_CK_0, H_CK_2, and H_CK_7. In the “H_CK_0” *vs*. “DH_CK_0” comparison group, there were 21,564 DEGs, including 10,042 upregulated genes and 6,990 downregulated genes. However, in the “H_CK_7” *vs*. “DH_CK_7” comparison group, there were up to 31,795 DEGs, including 21,753 upregulated genes and 7,015 downregulated genes (**Figures 2**, **3a**), suggesting that there are abundant DEGs during de-hulled and hulled seed germination.

Furthermore, a total of 3,550 and 1,132 DEGs were found in the three comparison groups (“H_CK_0” *vs*. “DH_CK_0”, “H_CK_2” *vs*. “DH_CK_2”, and “H_CK_7” *vs*. “DH_CK_7”), respectively (**Figures 3b, c**). In order to identify the expression patterns of DEGs in each comparison group, we performed a cluster analysis (**Figures 3d–f**).

### 3.3 GO and KEGG enrichment analysis

**Figure 4:**
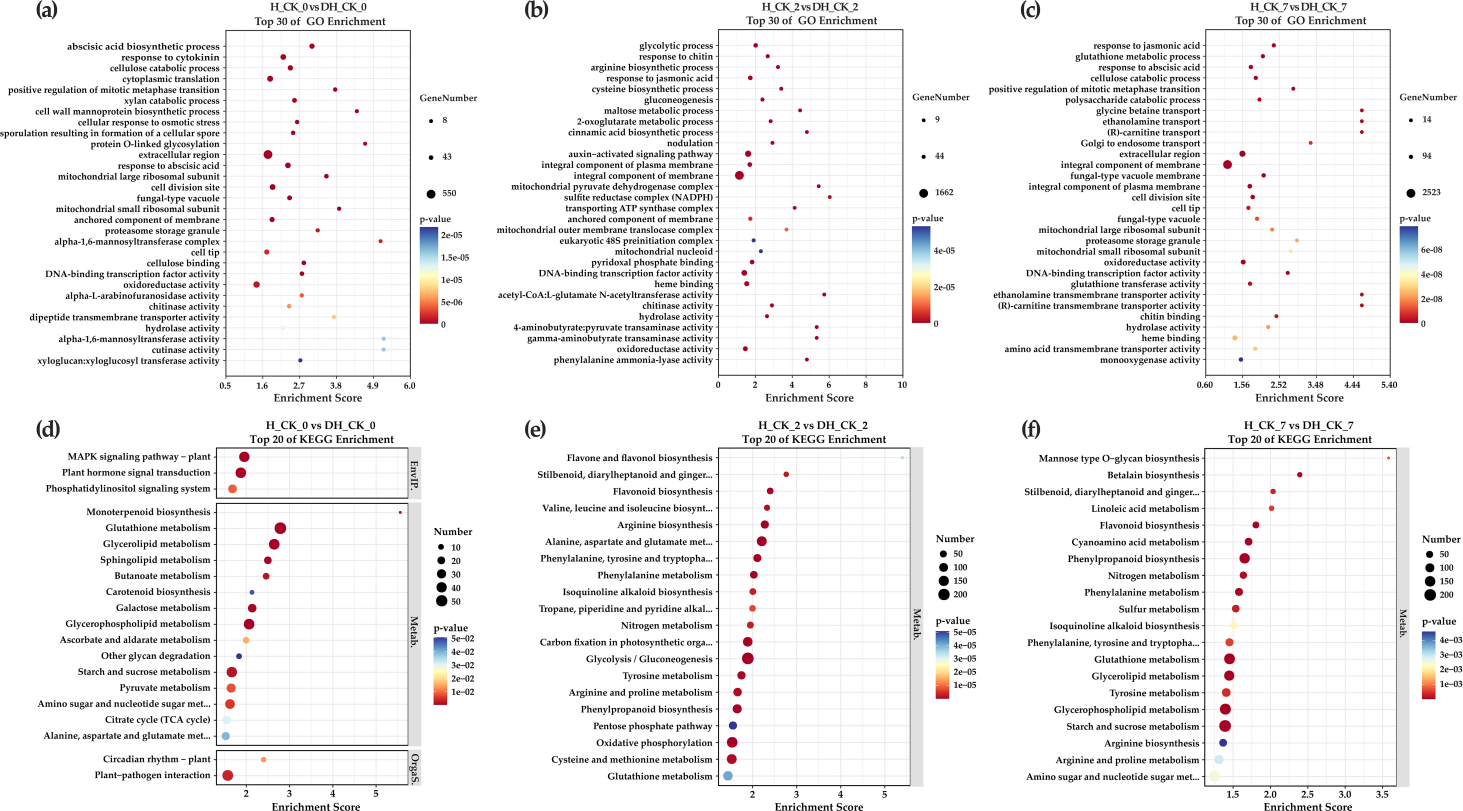
GO and KEGG functional pathways enrichment analysis of DEGs. **(a, d)** The comparison group H_CK_0 *vs*. DH_CK_0. **(b, e)** The comparison group H_CK_2 *vs*. DH_CK_2. **(c, f)** The comparison group H_CK_7 *vs*. DH_CK_7. DH_CK_0, DH_CK_2, and DH_CK_7 represented the de-hulled seeds treated with distilled water germinated for 0, 2, and 7 days, respectively. H_CK_0, H_CK_2, and H_CK_7 represented the hulled seeds treated with distilled water germinated for 0, 2, and 7 days, respectively.

Using GO enrichment analysis, the potential functions of DEGs in each comparison group were further divided into three categories and 30 enrichment terms. In the “H_CK_0” *vs*. “DH_CK_0” comparison group, cellulose catabolic process (GO:0030245, 88 DEGs), xylan catabolic process (GO:0045493, 53 DEGs), and DNA-binding transcription factor activity (GO:0000981, 41 DEGs) were significantly enriched (**Figure 4a**). In the “H_CK_2” *vs*. “DH_CK_2” comparison group, glycolytic process (GO:0006096, 106 DEGs), arginine biosynthetic process (GO:0006526, 35 DEGs), maltose metabolic process (GO:0000023, 15 DEGs), and DNA-binding transcription factor activity (GO:0003700, 354 DEGs) were significantly enriched (**Figure 4b**). In the “H_CK_7” *vs*. “DH_CK_7” comparison group, glutathione metabolic process (GO:0006749, 94 DEGs), cellulose catabolic process (GO:0030245, 99 DEGs), DNA-binding transcription factor activity (GO:0000981, 58 DEGs), and glutathione transferase activity (GO:0004364, 101 DEGs) were significantly enriched (**Figure 4c**), and these are all related to seed germination in plants.

Subsequently, we searched for the metabolic pathways involved in these DEGs through the KEGG database, and the results of the top 20 significant enrichment terms and pathways were shown. The results suggested that many metabolic pathways changed during the germination of de-hulled and hulled *L. chinensis* seeds. Excitingly, glutathione metabolism (ko00480, 54, 120, and 165 DEGs) was found in three comparison groups. Mitogen-activated protein kinase (MAPK) signaling pathway-plant (ko04016) and starch and sucrose metabolism (ko00500) were found in three comparison groups. In addition, MAPK signaling pathway-plant (ko04016, 41 DEGs) and plant hormone signal transduction (ko04075, 42 DEGs) were significantly enriched in the “H_CK_0” *vs*. “DH_CK_0” comparison group (**Figure 4d**). Starch and sucrose metabolism (ko00500, 41 and 202 DEGs) was significantly enriched in the “H_CK_0” *vs*. “DH_CK_0” and “H_CK_7” *vs*. “DH_CK_7” comparison groups (**Figures 4d, f**). Arginine and proline metabolism (ko00330, 91 and 98 DEGs) and arginine biosynthesis (ko00220, 81 and 66 DEGs) were significantly enriched in the “H_CK_2” *vs*. “DH_CK_2” and “H_CK_7” *vs*. “DH_CK_7” comparison groups, respectively (**Figures 4e, f**).

### 3.4 Effect of seed hulls on physiological and biochemical indicators

**Figure 5:**
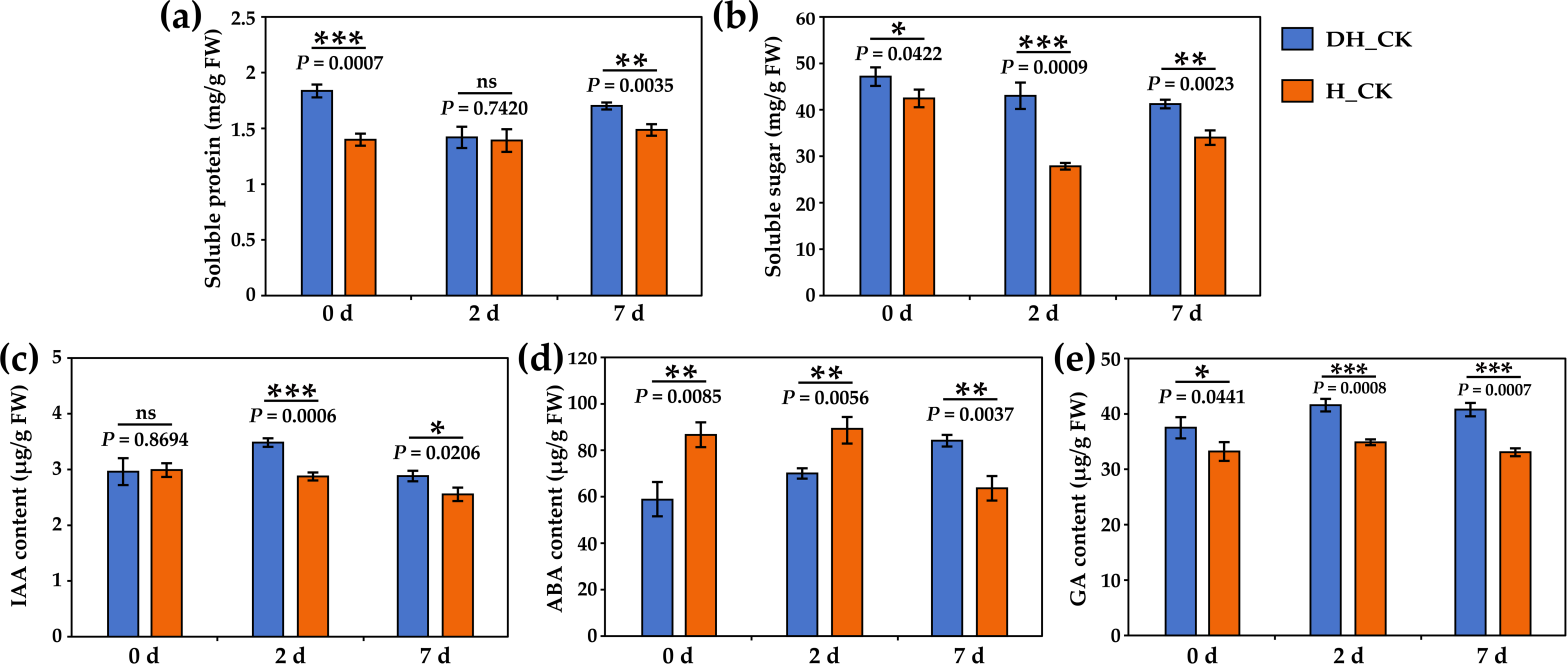
Effect of seed hulls on physiological and biochemical indicators during seed germination. **(a)** Soluble protein content. **(b)** Soluble sugar content. **(c)** IAA content. **(d)** ABA content. **(e)** GA content. 0d, 2d, and 7d represented the 0, 2, and 7 days of seed germination, respectively. DH, de-hulled seeds; H, hulled seeds. The data were retrieved from three biological replicates. Data are the mean ± SD of three biological repeats. ns, non-significant. *p<0.05, **p<0.01, ***p<0.001 (*t*-tests).

In order to explore the effect of seed hulls on physiological indexes during the germination of *L. chinensis* seeds, the content of soluble protein, soluble sugar, IAA, ABA, and GA, and the activity of α/β-amylase, lipase, and neutral protease were determined. With the seed absorbing water, the storage protein, starch, and sucrose stored in the embryo can be rapidly decomposed into soluble protein and soluble sugar, which accumulates energy for seed germination. Results showed that soluble protein and soluble sugar content of hulled seeds were significantly lower (*p*<0.05) during seed germination compared with the de-hulled seeds (**Figures 5a, b**). In addition, the IAA and GA content of hulled seeds was significantly (*p*<0.05) decreased on the 2nd and 7th day of seed germination compared with the de-hulled seeds (**Figures 5c, e**). Furthermore, the ABA content of hulled seeds was significantly (*p*<0.05) increased on the 0th and 2nd day of seed germination compared with the de-hulled seeds (**Figure 5d**).

**Figure 6:**
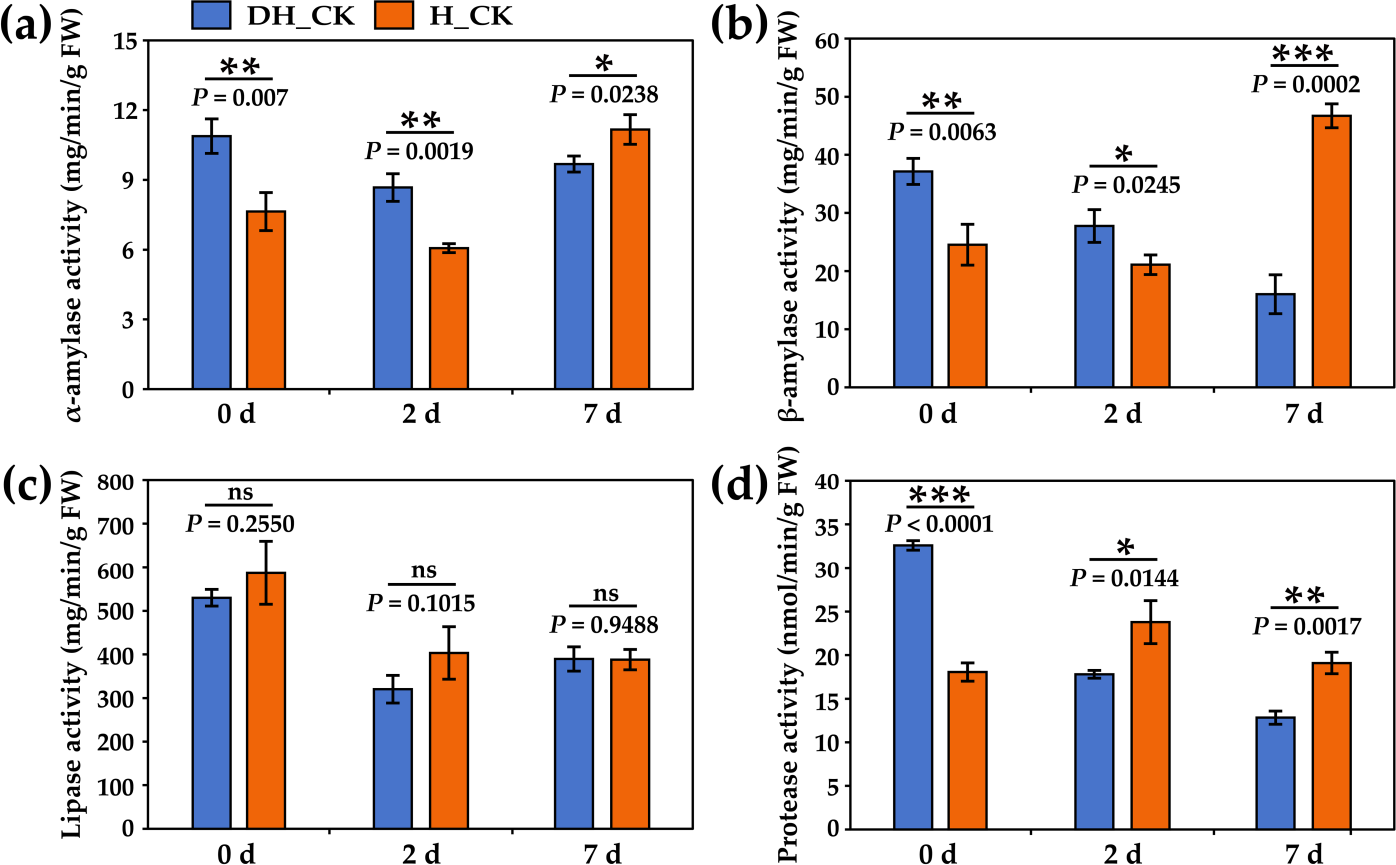
Effect of seed hulls on enzyme activity during seed germination. **(a)** α-amylase activity. **(b)** β-amylase activity. **(c)** Lipase activity. **(d)** Neutral protease activity. 0d, 2d, and 7d represented the 0, 2, and 7 days of seed germination, respectively. DH, de-hulled seeds; H, hulled seeds. The data were retrieved from three biological replicates. Data are the mean ± SD of three biological repeats. ns, non-significant. *p<0.05, **p<0.01, ***p<0.001 (*t*-tests).

Amylase, lipase, and neutral protease can decompose the energy storage substances starch, lipid, and protein, and then provide energy for seed germination. The results showed that the activity of α/β-amylase of de-hulled seeds was significantly increased (*p*<0.05) on the 0th and 2nd day of seed germination compared with the hulled seeds (**Figures 6a, b**). No significant change in lipase activity was found in de-hulled and hulled seeds during seed germination (**Figure 6c**). Furthermore, neutral protease activity in de-hulled seeds was significantly higher (*p*<0.0001) on the 0th day of seed germination than that in hulled seeds (**Figure 6d**). These results indicated that seed hulls might hinder seed germination by inhibiting enzyme activity.

### 3.5 RNA-seq validation using qRT-PCR

**Figure 7:**
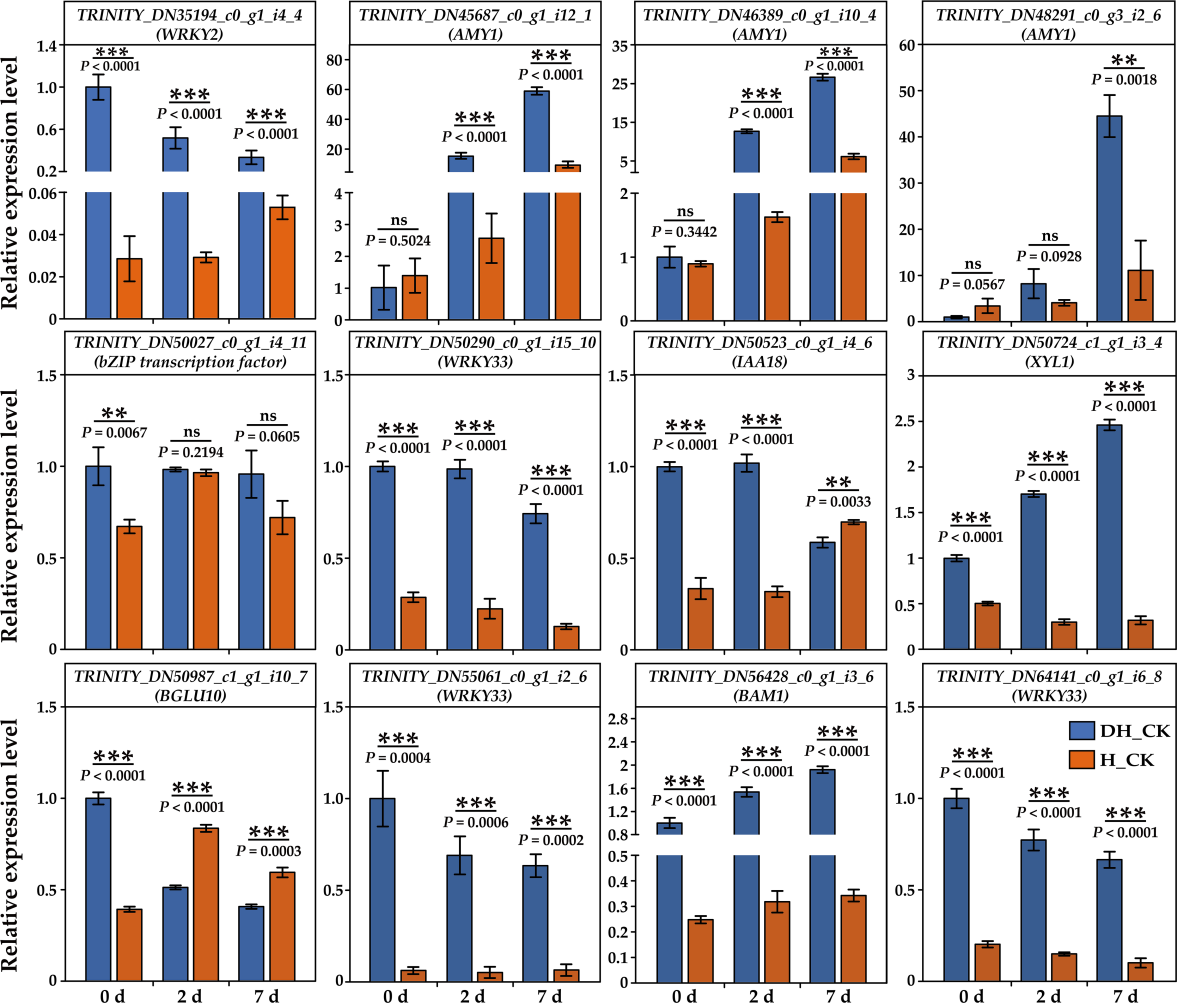
Validation of the relative expression levels of 12 selected DEGs by RT-qPCR, which was calculated as folds relative to DH_CK-0d. 0d, 2d, and 7d represented the 0, 2, and 7 days of seed germination, respectively. DH, de-hulled seeds; H, hulled seeds. The data were retrieved from three biological replicates. Data are the mean ± SD of three biological repeats. ns, non-significant. **p<0.01, ***p<0.001 (*t*-tests).

In this study, we selected 12 genes (including *WRKY2*/*33*, *AMY1*, *bZIP*, *IAA18*, *XYL1*, *BGLU10*, and *BAM1*) in the three comparison groups for qRT-PCR validation. Based on the results of qRT-PCR, we found that the expressions of *WRKY2*, *WRKY33*, *XYL1*, and *BAM1* in de-hulled seeds were significantly (*p*<0.01) higher than those in hulled seeds during seed germination (**Figure 7**). Two *AMY1* genes were highly expressed in de-hulled seeds at 2 and 7 days of germination. The results showed that the expression patterns of these genes were generally consistent with the trends presented in the RNA-seq data, validating the accuracy and reliability of the RNA-seq data.

### 3.6 Identification of key genes and modules during seed germination by WGCNA

**Figure 8:**
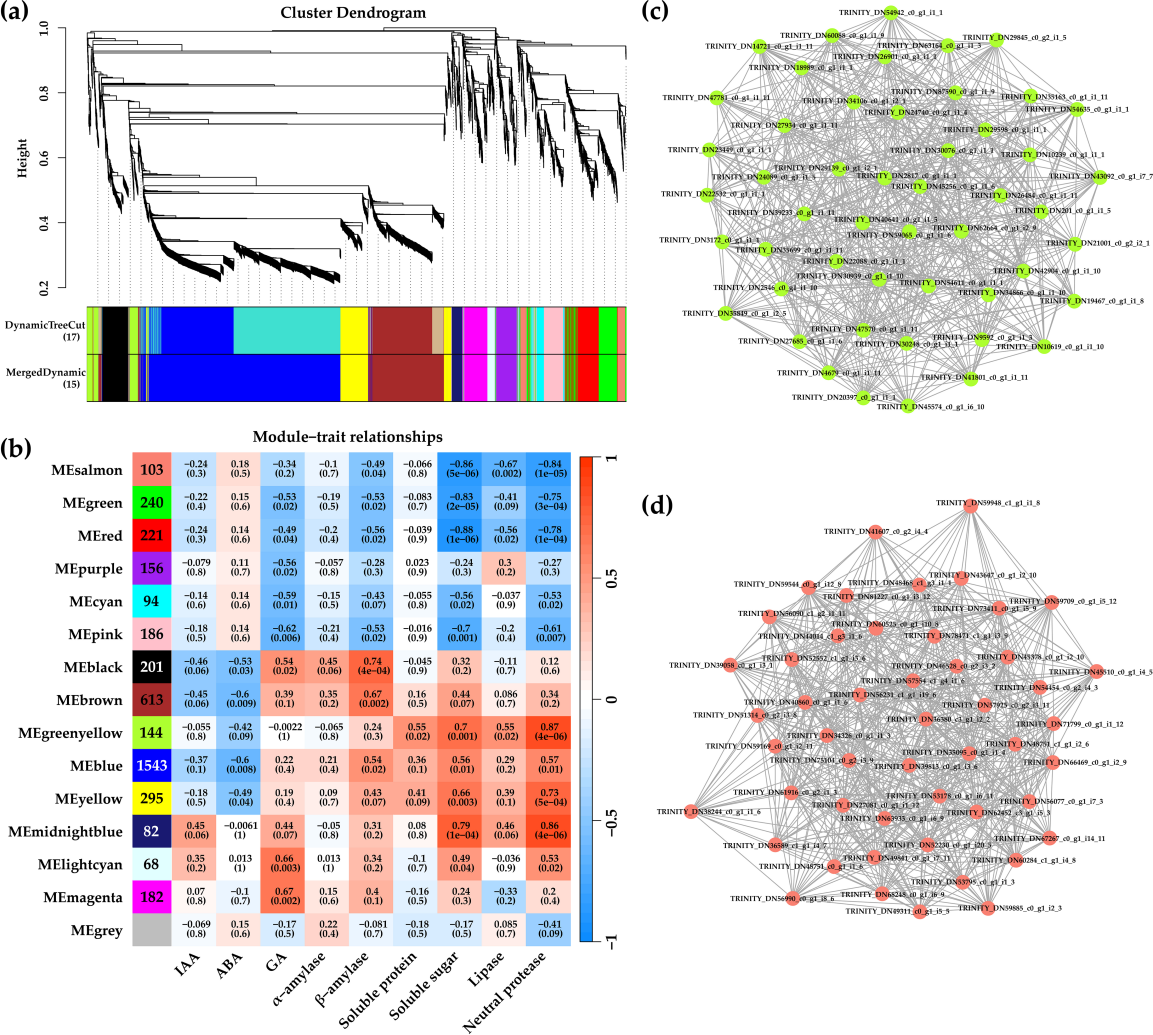
Analysis results of WGCNA. **(a)** Hierarchical clustering tree showing co-expression modules identified by WGCNA. **(b)** Module sample association relationships. **(c)** Correlation networks of hub genes in the yellow module. **(d)** Heatmap of hub genes in the yellow module.

The WGCNA method was used to explore the different physiological parameters and key genes during the germination of de-hulled and hulled seeds. In WGCNA, modules were defined as clusters of highly correlated genes, and genes within the same module had high correlation coefficients. A total of 4,682 genes were included in 15 modules, among which 554 genes were in the gray module (**Figure 8a**). The gray module is the gene set that cannot be assigned to any module, without reference significance. This study carefully studied the genes in each module, from 68 genes in the light cyan module to 1,543 genes in the blue module. Furthermore, the physiological indexes of de-hulled and hulled seed germination were associated with modules, and their correlation was revealed (**Figure 8b**; **Supplementary Figure S3**). The green–yellow and salmon modules were shown as representatives. The genes in the green–yellow module were positively correlated with soluble protein, soluble sugar, lipase, and neutral protease (*p*<0.05). The genes in the salmon module were negatively correlated with soluble sugar, lipase, and neutral protease (*p*<0.001). According to the edge weight ≥ 0.02, 50 key genes were screened in the green–yellow module and the salmon module, respectively (**Figures 8c, d**). The results suggested that these genes might be involved in seed hull inhibition at different germination stages of *L. chinensis* seeds.

### 3.7 Diagram of the regulatory network

**Figure 9:**
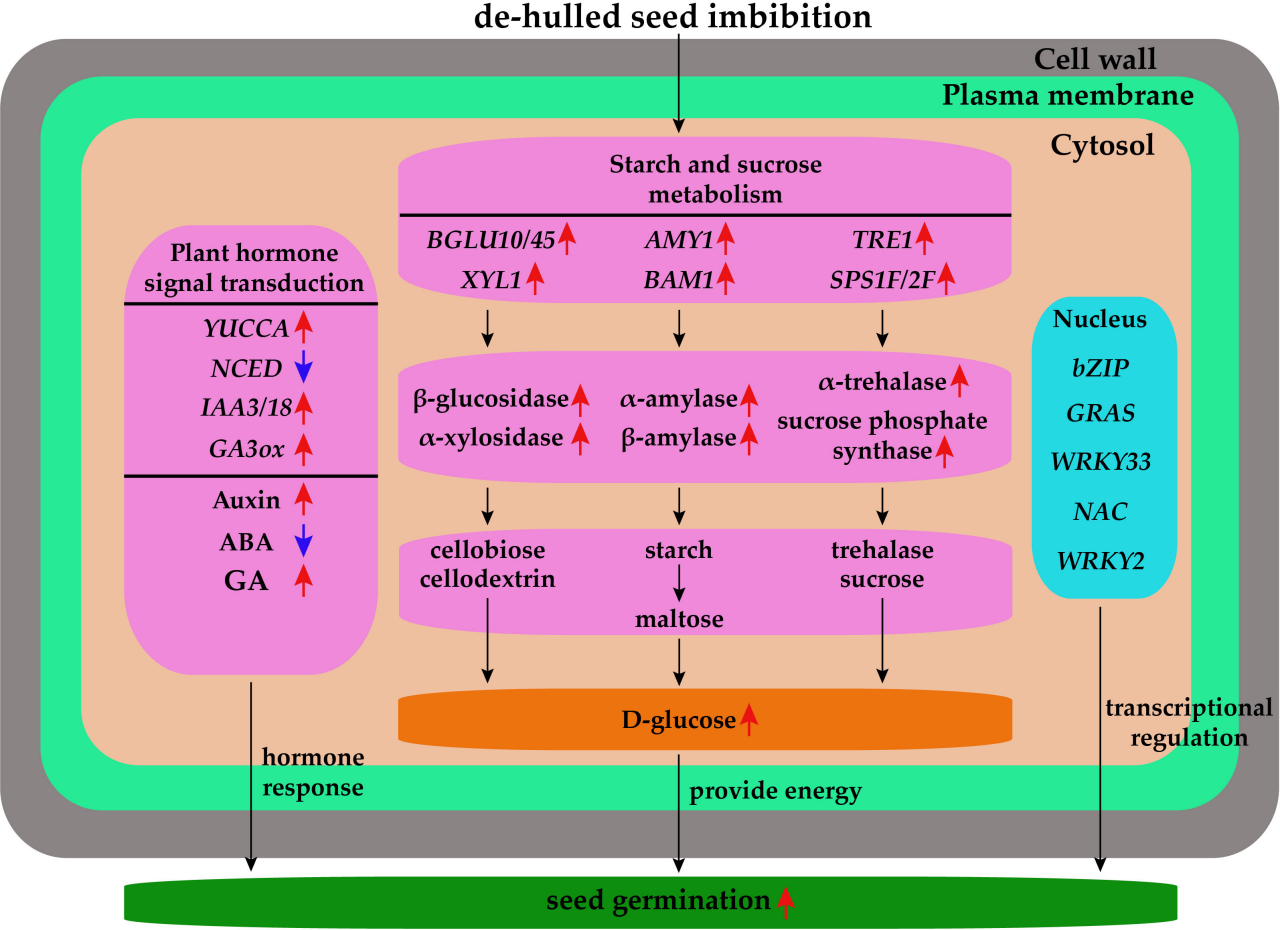
The regulatory network diagram of the effect of seed hulls on the seed germination of *L. chinensis*. The red arrows represent upregulation, and the blue arrows represent downregulation.

A regulatory network diagram was constructed according to the DEGs and metabolites of de-hulled and hulled seeds during germination after water absorption (**Figure 9**). The de-hulled seeds after water absorption could break seed dormancy faster and promote germination through plant hormone signal transduction, starch and sucrose metabolism, and transcriptional regulation. In plant hormone signal transduction, the expressions of *YUCCA*, *IAA3*/*18*, and *GA3ox* were upregulated while NCED was downregulated, resulting in the increase of auxin and GA content and the decrease of ABA content. In starch and sucrose metabolism, the expressions of *BGLU10*/*45*, *XYL1*, *AMY1*, *BAM1*, *TRE1*, and *SPS1F*/*2F* were upregulated. Differential expressions of these genes promoted the synthesis of β-glucosidase, α-xylosidase, α-amylase, β-amylase, α-trehalase, and sucrose phosphate synthase. Subsequently, these hydrolases catalyze the generation of glucose from starch, trehalose, and cellodextrin and ultimately provide energy for *L. chinensis* seed germination. In addition, de-hulled and hulled seeds after water absorption also changed the expression levels of many TFs encoding genes, including *bZIP*, *GRAS*, *WRKY33*, *NAC*, and *WRKY2*.

## 4 Discussion

It is widely accepted that seed dormancy and germination are precisely regulated by diverse endogenous plant hormones (Koornneef et al., 2002). Studies have found that seed hulls contain substances that affect hormone synthesis, such as phenols and aldehydes. In addition, ABA and GA are two major plant hormones that play key roles in regulating seed dormancy and germination (Shu et al., 2016). Recent studies on seed germination have focused on the model species *Arabidopsis*, rice, wheat, and tobacco (Gao et al., 2012; Agacka-Mołdoch et al., 2021; Hao et al., 2021). However, the reports on the inhibition of seed germination by seed hulls mainly focus on physiological mechanisms, and there are few studies on the molecular mechanisms by which seed hulls regulate the seed germination of *L. chinensis* at the transcriptional level (Li et al., 2021). To explore whether the seed hulls affect the germination of *L. chinensis* seeds via plant hormones (such as IAA, ABA, and GA) and other metabolic pathways, we used sterile water to treat de-hulled and hulled seeds and measured germination rate and other indicators. The results showed that seed hulls could significantly inhibit the germination of *L. chinensis* seeds (**Figures 1**, **5**).

Seed germination is a key and complex process in the plant life cycle, which is precisely regulated by multiple genes. With suitable conditions, the metabolic process of seeds is activated rapidly. After absorbing water, nutrients stored in seeds are released under the regulation of many genes and provide essential nutrition and energy for seed germination and development (Wolny et al., 2018). In this study, we performed RNA-seq analysis of de-hulled and hulled seeds treated with distilled water at different germination times, and more than 50,000 genes were generated (**Figures 2**, **3**). Previously, many genes in the plant hormone signal transduction pathway were identified as being involved in regulating seed germination (Liao et al., 2019). Similarly, GO annotation results showed that the ABA biosynthetic process (GO:0009688), response to cytokinin (GO:0009735), response to jasmonic acid (GO:0009753), auxin-activated signaling pathway (GO:0009734), and response to ABA (GO:0009737) were significantly enriched in the seed germination process (**Figure 4**), indicating that these pathways play a key role in seed hulls affecting seed germination. The KEGG pathway analysis showed that the plant hormone signal transduction (ko04075) was a significantly enriched pathway (**Figure 4**). In addition, a previous study has shown that overexpression of *IAA8* promotes seed germination by inhibiting *ABI3* expression (Hussain et al., 2020). Similarly, we found that the *ABI2* homologous gene *HAB2* was significantly downregulated in de-hulled seeds, while *IAA3* and *IAA18* were significantly upregulated in this study (**Supplementary Table S3**). Moreover, the content of IAA in de-hulled seeds was significantly higher than that in hulled seeds (**Figure 5c**). Therefore, we speculated that *ABI2* homologous upregulation and *IAA3* and *IAA18* downregulation in hulled seeds might inhibit seed germination by affecting the ABA signaling pathway of *L. chinensis*.

The seed hulls, as a critical physical barrier regulating seed dormancy and germination, exert dual roles in protecting the embryo and modulating environmental cues through their mechanical properties and chemical composition. Mechanically, the rigidity and impermeability of seed hulls can restrict gas exchange and water imbibition, thereby delaying or inhibiting germination initiation (Debeaujon et al., 2000). Biochemical factors, including phenolic compounds, ABA, and germination inhibitors, further contribute to dormancy maintenance by suppressing embryonic growth or altering hormonal balance. In addition, previous studies have shown that organic acids, esters, and alcohols are ubiquitously distributed in seed hulls, where they may act as germination inhibitors by lowering pH or scavenging reactive oxygen species (ROS) (Bailly et al., 2008). In this study, KEGG analysis showed that some DEGs were enriched in arginine and proline metabolic pathways (**Figures 4e, f**), which are important precursors for the synthesis of organic acids.

Starch and sucrose, as the main energy storage substances in seeds, are usually stored in the endosperm or cotyledons in a granular form (Li et al., 2013). During seed germination, starch and sucrose are hydrolyzed into soluble sugars such as glucose and maltose under the action of hydrolases, which provide energy for seed germination. Amylase is generally classified into α-amylase and β-amylase. Our results showed that many genes were enriched in the starch and sucrose metabolic (ko00500) pathway (**Figure 4**). We observed that genes associated with amylase, including *AMY1* and *BAM1*, were significantly upregulated in de-hulled seeds (**Supplementary Table S4**). In the process of starch metabolism, these genes catalyze the hydrolysis of glycogen, such as starch, and provide more energy for seed germination. Furthermore, the activity of α-amylase and β-amylase in de-hulled seeds was also significantly higher than that in hulled seeds (**Figures 6a, b**). Two β-glucosidase coding genes (*BGLU10* and *BGLU45*) and one α-xylosidase coding gene (*XYL1*) were found to be significantly upregulated in de-hulled seeds (**Supplementary Table S4**), promoting glucose synthesis. These results showed that seed hulls could inhibit the seed germination of *L. chinensis* by regulating starch and sucrose metabolism.

The MAPK signaling pathway is a signal transduction pathway that transduces extracellular stimulation signals into cells. It converts external stimulation signals into a series of intracellular physiological reactions by activating a series of protein kinase cascades (Cargnello and Roux, 2011; Kyriakis and Avruch, 2012). In addition, the MAPK signaling pathway can promote the water absorption process of seeds and subsequent water regulation, providing necessary water conditions for seed germination (Danquah et al., 2014; Tian et al., 2021). It was reported that the activation of MKK3 could promote seed germination by inhibiting ABA biosynthesis enzyme gene expression and inducing expression of GA biosynthesis enzyme genes (Otani et al., 2024). In the current study, several genes were enriched in the MAPK signaling pathway (ko04016) (**Figure 4**). Furthermore, the expression of *MKK3* and *WRKY* was significantly downregulated in hulled seeds (**Supplementary Table S5**), suggesting that seed hulls inhibited the seed germination of *L. chinensis* by regulating the MAPK signaling pathway.

It is well known that biological processes in plants are carried out by a complex regulatory network of TFs, such as AP2/ERF, WRKY, bHLH, bZIP, MYB, and NAC (Gaudinier and Brady, 2016; Meraj et al., 2020). It was reported that ERF1 plays important roles in regulating abiotic stresses and primary root elongation in *Arabidopsis* (Mao et al., 2016). In addition, AtbZIP44 positively regulates *Arabidopsis* seed germination, and the knock-out lines of *AtbZIP44* have a significantly slower germination than the wild type (Iglesias-Fernández et al., 2013). Notably, AP2/ERF and bZIP TFs were identified by KEGG enrichment analysis in this study (**Supplementary Table S3**), indicating that these TFs might be involved in the plant hormone-mediated seed germination of *L. chinensis* with or without seed hulls. NAC TFs are one of the largest plant-specific TF families and play significant roles in plant growth, development, and response to environmental stresses (Shao et al., 2015). *TaNAC019-A1*, a homologous gene of *Arabidopsis NAC019*, negatively regulates starch synthesis during wheat endosperm development (Liu et al., 2020). In the present study, *NAC019* showed a higher expression level during the germination of de-hulled *L. chinensis* seed (**Supplementary Table S6**). Furthermore, the results of WGCNA showed that *NAC019* was co-expressed with many *AMY* genes (**Figure 8**; **Supplementary Table S6**), indicating that it might promote the seed germination of *L. chinensis* by regulating starch metabolism.

## 5 Conclusions

In this study, we performed transcriptomic analysis to analyze the potential regulatory factors for seed hulls that affect the seed germination of *L. chinensis*. It was found that the DEGs related to plant hormone signal transduction, starch and sucrose metabolism, and MAPK signaling pathway might play important roles in the seed germination of *L. chinensis*. Combined with WGCNA and enrichment analysis, WRKY, AP2/ERF, bZIP, and NAC TFs were identified as potential essential TFs for seed hulls affecting *L. chinensis* seed germination. These results provide valuable genetic resources for studying the molecular mechanism of *L. chinensis* seed germination and lay a foundation for future molecular breeding research with a high germination rate.

## Data Availability

The original contributions presented in the study are included in the article/**Supplementary Material**. Further inquiries can be directed to the corresponding author.

## Supplementary material

Supplementary Figure 1 The PCA and correlation analysis of transcriptome data.

Supplementary Figure 2 Analysis of the expression trend of DEGs.

Supplementary Figure 3 The network diagram generated by cytoscape software.

Supplementary Figure 4 GO and KEGG analysis of genes from the yellow-green module.

Supplementary Table 1 The primers in this study.

Supplementary Table 2 Statistical analysis of the reads.

Supplementary Table 3 The DEGs involved in plant hormone signaling pathways.

Supplementary Table 4 The DEGs involved in starch and sucrose metabolism.

Supplementary Table 5 The DEGs involved in MAPK signaling pathway.

Supplementary Table 6 The analysis of WGCNA.
