# Supplementary figures and images for: Integrated transcriptomics and physio-biochemical analysis revealed key genes affecting the seed germination of Leymus chinensis
Source: Front Plant Sci. 2025 Oct 31;16:1696194. doi: 10.3389/fpls.2025.1696194 (PMC12615476; doi:10.3389/fpls.2025.1696194)

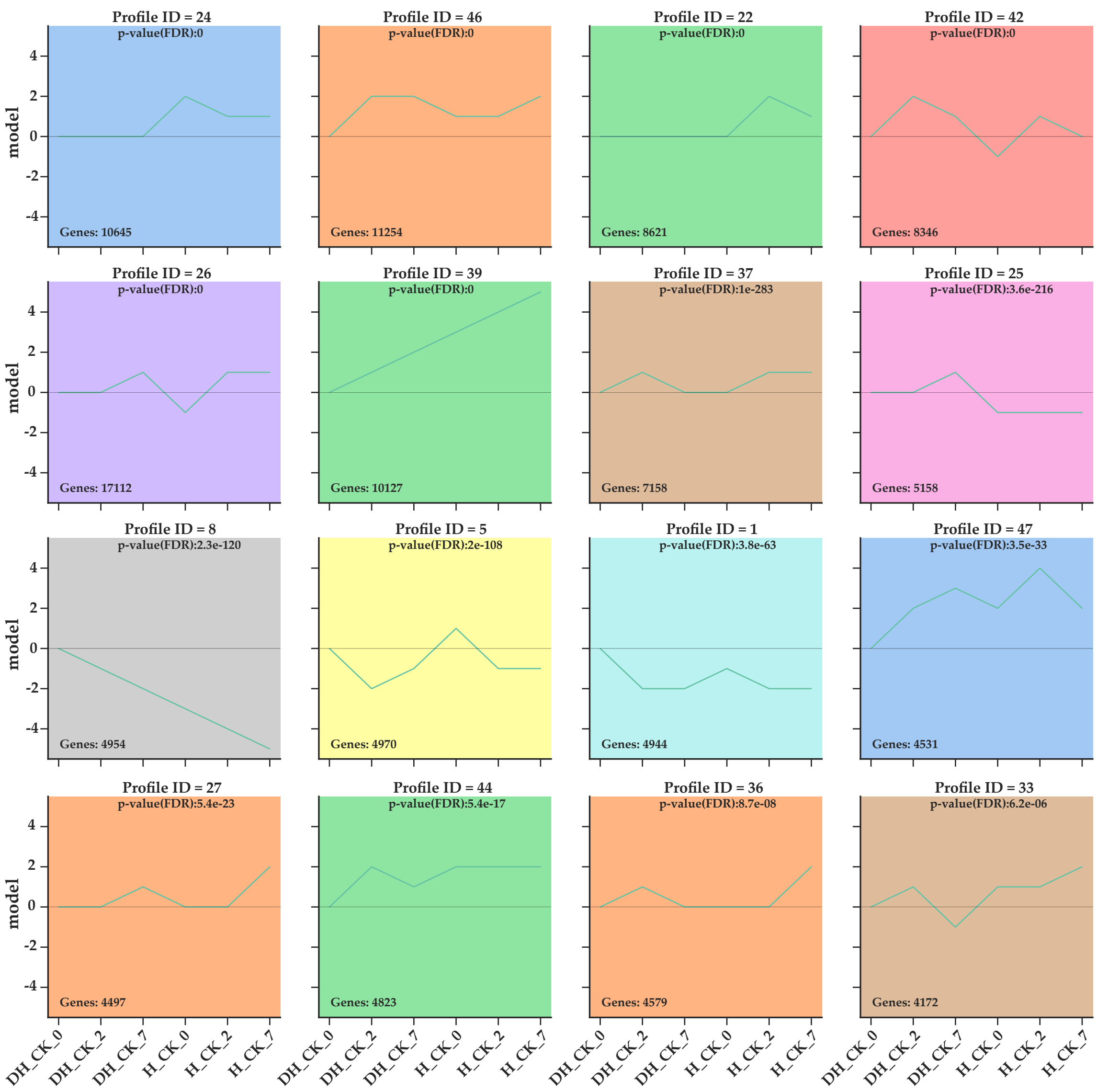

Supplement: Supplementary Figure 1 — The PCA and correlation analysis of transcriptome data. [file DataSheet1.zip › Supplementary Materials/Supplementary Figure S2.pdf]

(a) MEblack

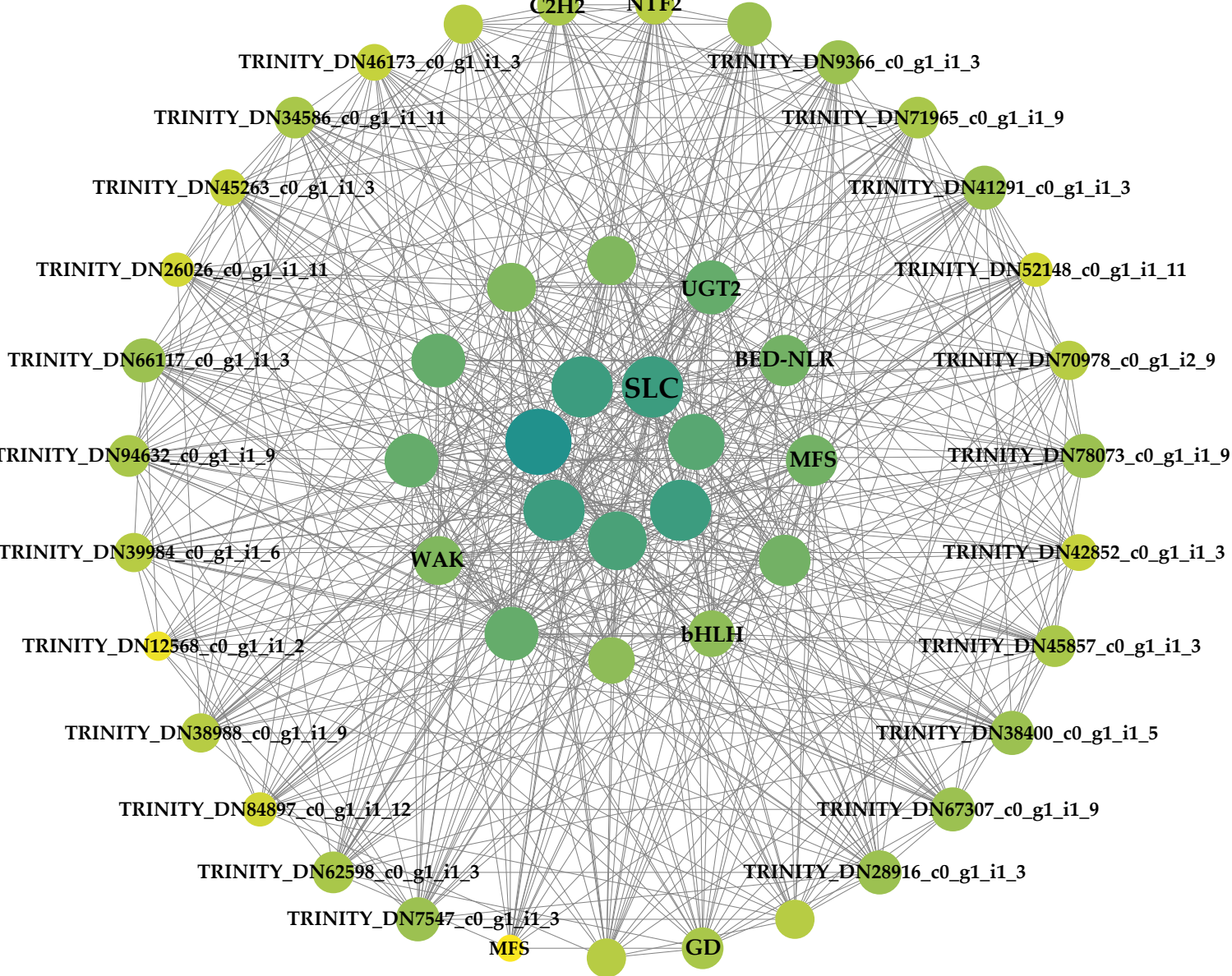

(b) MEblue

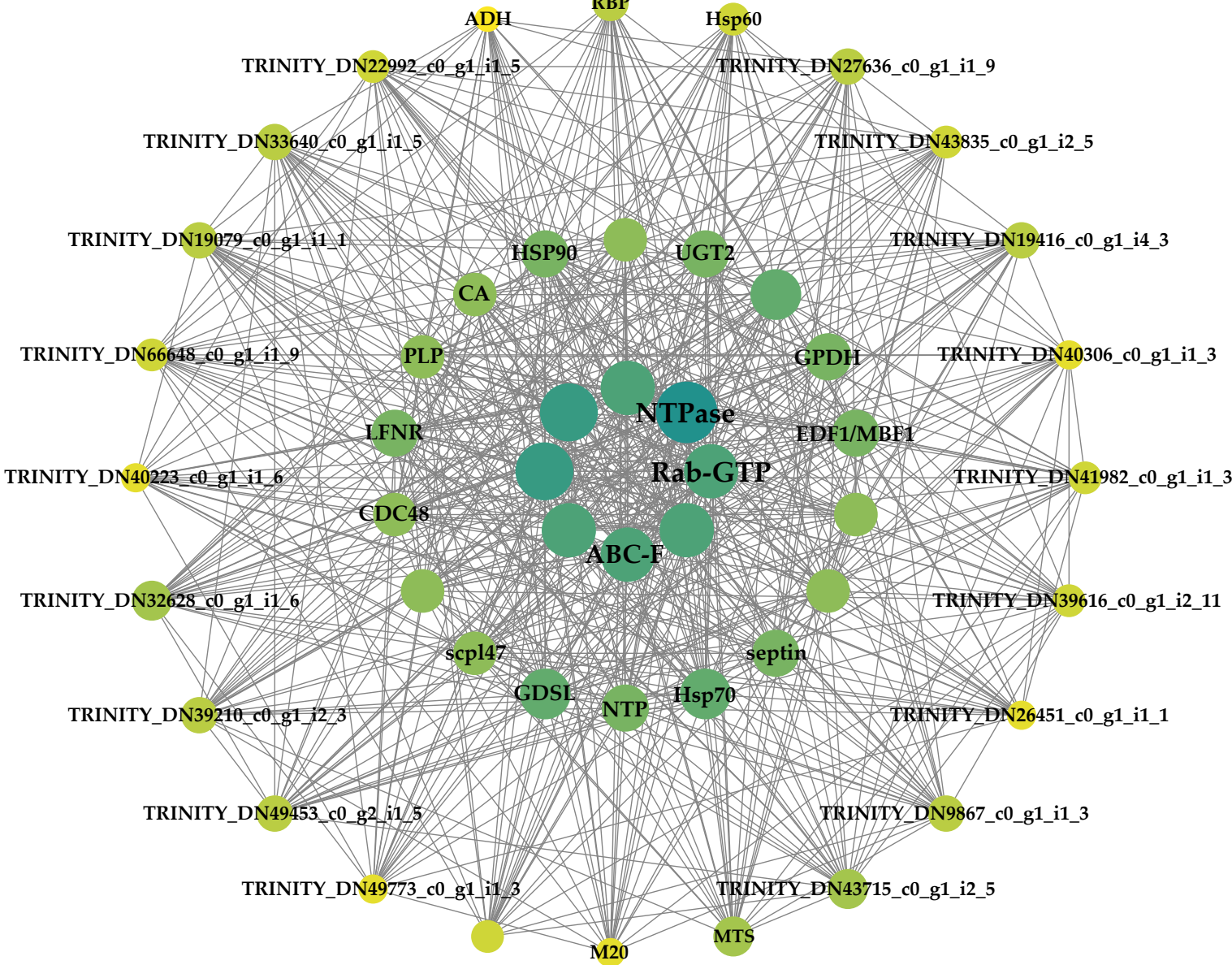

(c) MEbrown

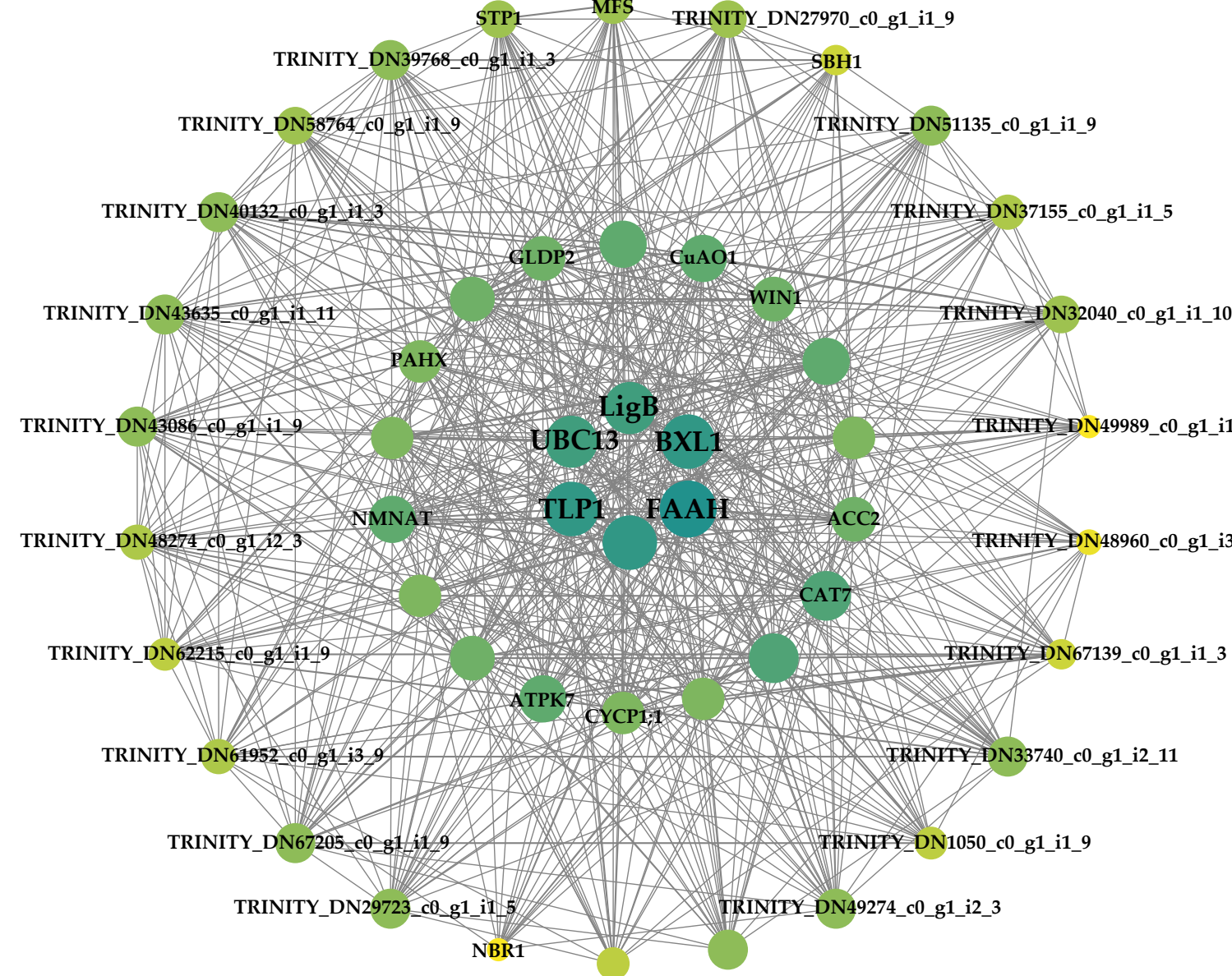

(d) MEgreen

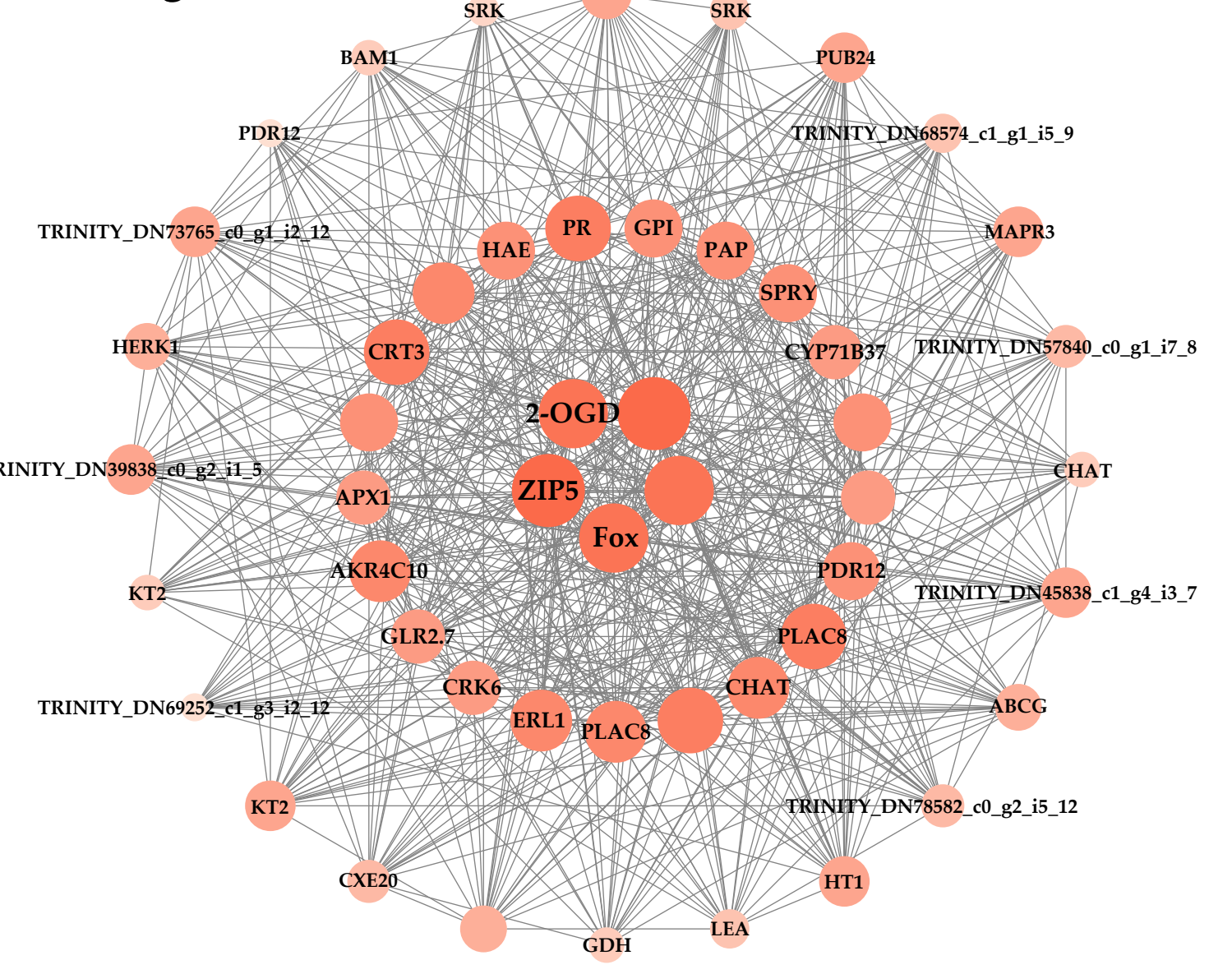

(e) MEgreenyellow

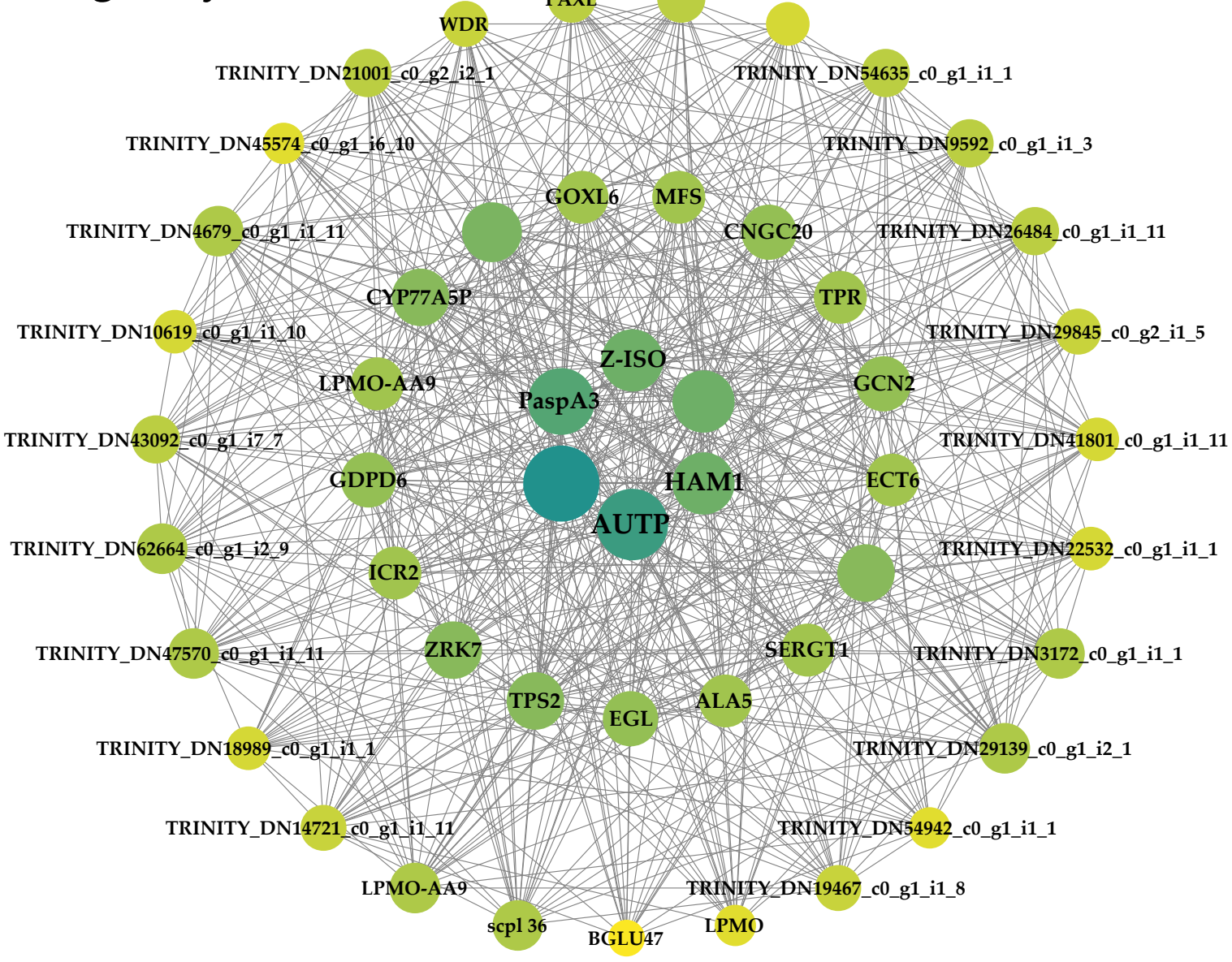

(f) MEsalmon

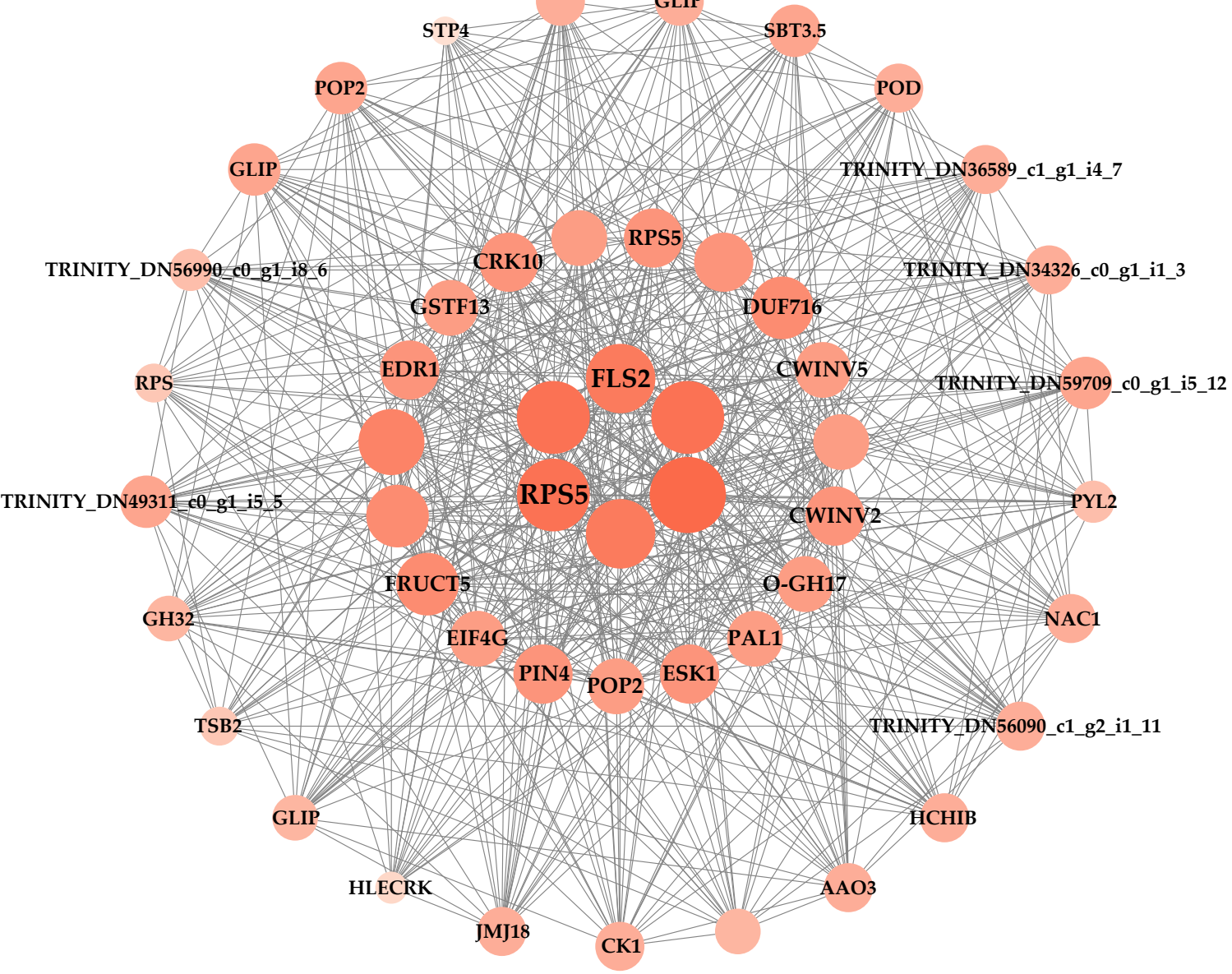

Supplement: Supplementary Figure 1 — The PCA and correlation analysis of transcriptome data. [file DataSheet1.zip › Supplementary Materials/Supplementary Figure S3.pdf]
