# Supplementary material for: Integrated transcriptomics and physio-biochemical analysis revealed key genes affecting the seed germination of Leymus chinensis
Source: Front Plant Sci. 2025 Oct 31;16:1696194. doi: 10.3389/fpls.2025.1696194 (PMC12615476; doi:10.3389/fpls.2025.1696194)
Supplement: Supplementary Figure 1 — The PCA and correlation analysis of transcriptome data. [file Supplementary Figure S1.pdf › Supplementary Materials/Supplementary Figure S1.pdf]

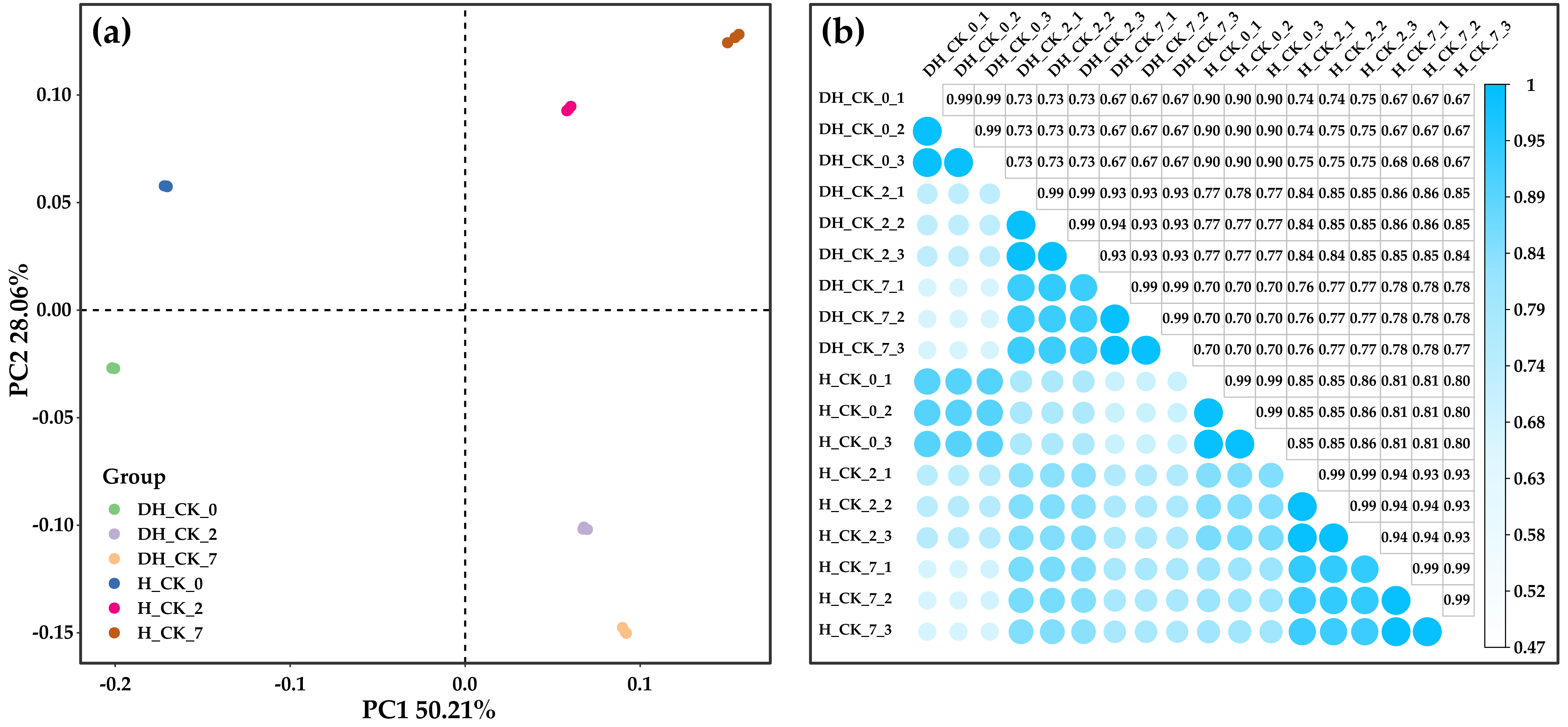

(a)
0.10
0.05
0.00
-0.05
-0.10
-0.15
PC2 28.06%
-0.2
-0.1
0.0
0.1
PC1 50.21%
Group
DH_CK_0
DH_CK_2
DH_CK_7
H_CK_0
H_CK_2
H_CK_7
(b)
DH_CK_0_1 DH_CK_0_2 DH_CK_0_3 DH_CK_2_1 DH_CK_2_2 DH_CK_2_3 DH_CK_7_1 DH_CK_7_2 DH_CK_7_3 H_CK_0_1 H_CK_0_2 H_CK_0_3 H_CK_2_1 H_CK_2_2 H_CK_2_3 H_CK_7_1 H_CK_7_2 H_CK_7_3
DH_CK_0_1 0.99 0.99 0.73 0.73 0.73 0.67 0.67 0.67 0.90 0.90 0.90 0.74 0.74 0.75 0.67 0.67 0.67
DH_CK_0_2 0.99 0.73 0.73 0.73 0.67 0.67 0.67 0.90 0.90 0.90 0.74 0.75 0.75 0.67 0.67 0.67
DH_CK_0_3 0.73 0.73 0.73 0.67 0.67 0.67 0.90 0.90 0.90 0.75 0.75 0.75 0.68 0.68 0.67
DH_CK_2_1 0.99 0.99 0.93 0.93 0.93 0.77 0.78 0.77 0.84 0.85 0.85 0.86 0.86 0.85
DH_CK_2_2 0.99 0.94 0.93 0.93 0.77 0.77 0.77 0.84 0.85 0.85 0.86 0.86 0.85
DH_CK_2_3 0.93 0.93 0.93 0.77 0.77 0.77 0.84 0.84 0.85 0.85 0.85 0.84
DH_CK_7_1 0.99 0.99 0.70 0.70 0.70 0.76 0.77 0.77 0.78 0.78 0.78
DH_CK_7_2 0.99 0.70 0.70 0.70 0.76 0.77 0.77 0.78 0.78 0.78
DH_CK_7_3 0.70 0.70 0.70 0.76 0.77 0.77 0.78 0.78 0.77
H_CK_0_1 0.99 0.99 0.85 0.85 0.86 0.81 0.81 0.80
H_CK_0_2 0.99 0.85 0.85 0.86 0.81 0.81 0.80
H_CK_0_3 0.85 0.85 0.86 0.81 0.81 0.80
H_CK_2_1 0.99 0.99 0.94 0.93 0.93
H_CK_2_2 0.99 0.94 0.94 0.93
H_CK_2_3 0.94 0.94 0.93
H_CK_7_1 0.99 0.99
H_CK_7_2 0.99
H_CK_7_3
1
0.95
0.89
0.84
0.79
0.74
0.68
0.63
0.58
0.52
0.47
