# Supplementary material for: Integrated transcriptomics and physio-biochemical analysis revealed key genes affecting the seed germination of Leymus chinensis
Source: Front Plant Sci. 2025 Oct 31;16:1696194. doi: 10.3389/fpls.2025.1696194 (PMC12615476; doi:10.3389/fpls.2025.1696194)
Supplement: Supplementary Figure 1 — The PCA and correlation analysis of transcriptome data. [file Supplementary Figure S4.pdf › Supplementary Materials/Supplementary Figure S4.pdf]

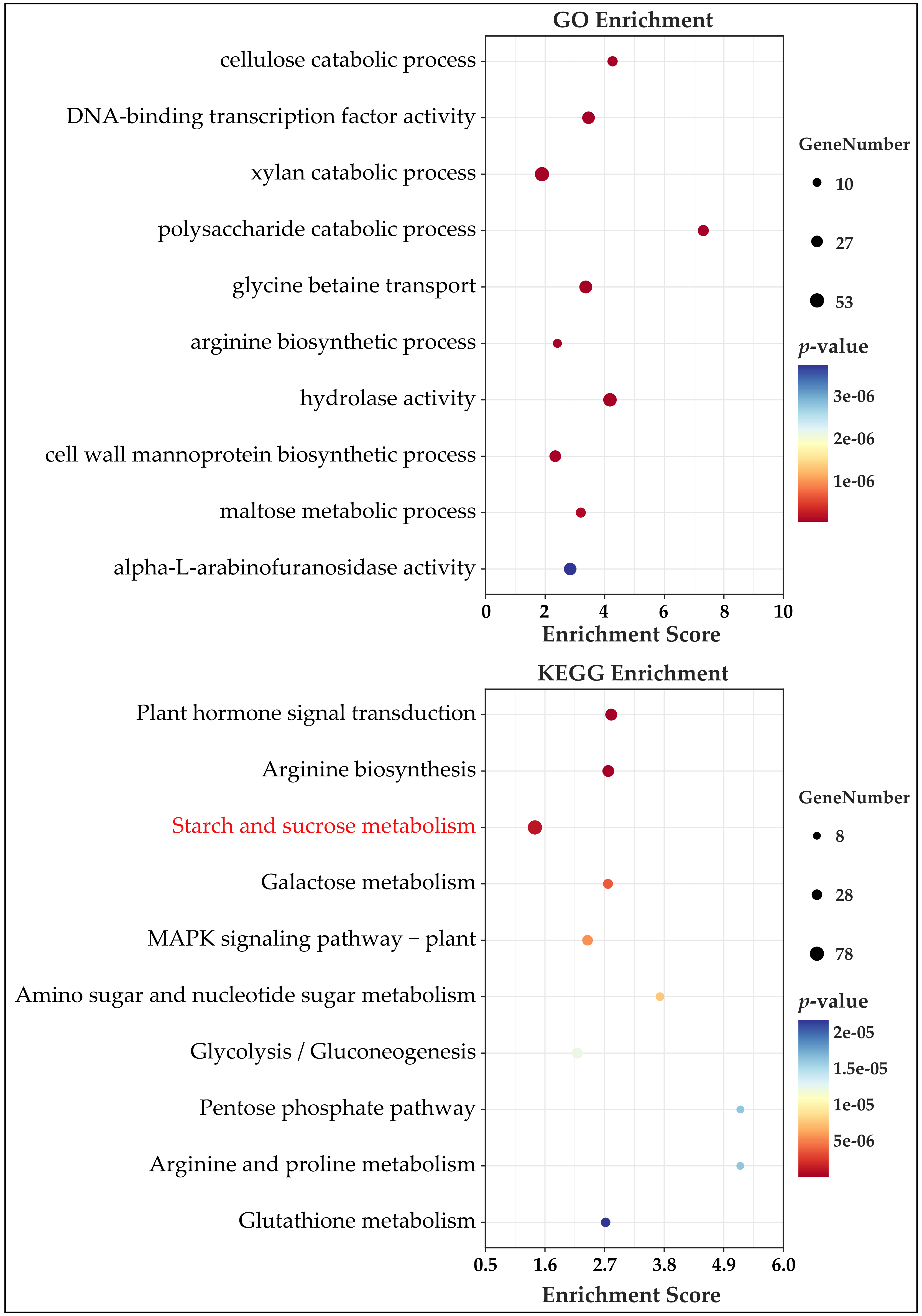

GO Enrichment
cellulose catabolic process
DNA-binding transcription factor activity
xylan catabolic process
polysaccharide catabolic process
glycine betaine transport
arginine biosynthetic process
hydrolase activity
cell wall mannoprotein biosynthetic process
maltose metabolic process
alpha-L-arabinofuranosidase activity
0 2 4 6 8 10
Enrichment Score
GeneNumber
10
27
53
p-value
3e-06
2e-06
1e-06
KEGG Enrichment
Plant hormone signal transduction
Arginine biosynthesis
Starch and sucrose metabolism
Galactose metabolism
MAPK signaling pathway − plant
Amino sugar and nucleotide sugar metabolism
Glycolysis / Gluconeogenesis
Pentose phosphate pathway
Arginine and proline metabolism
Glutathione metabolism
0.5 1.6 2.7 3.8 4.9 6.0
Enrichment Score
GeneNumber
8
28
78
p-value
2e-05
1.5e-05
1e-05
5e-06

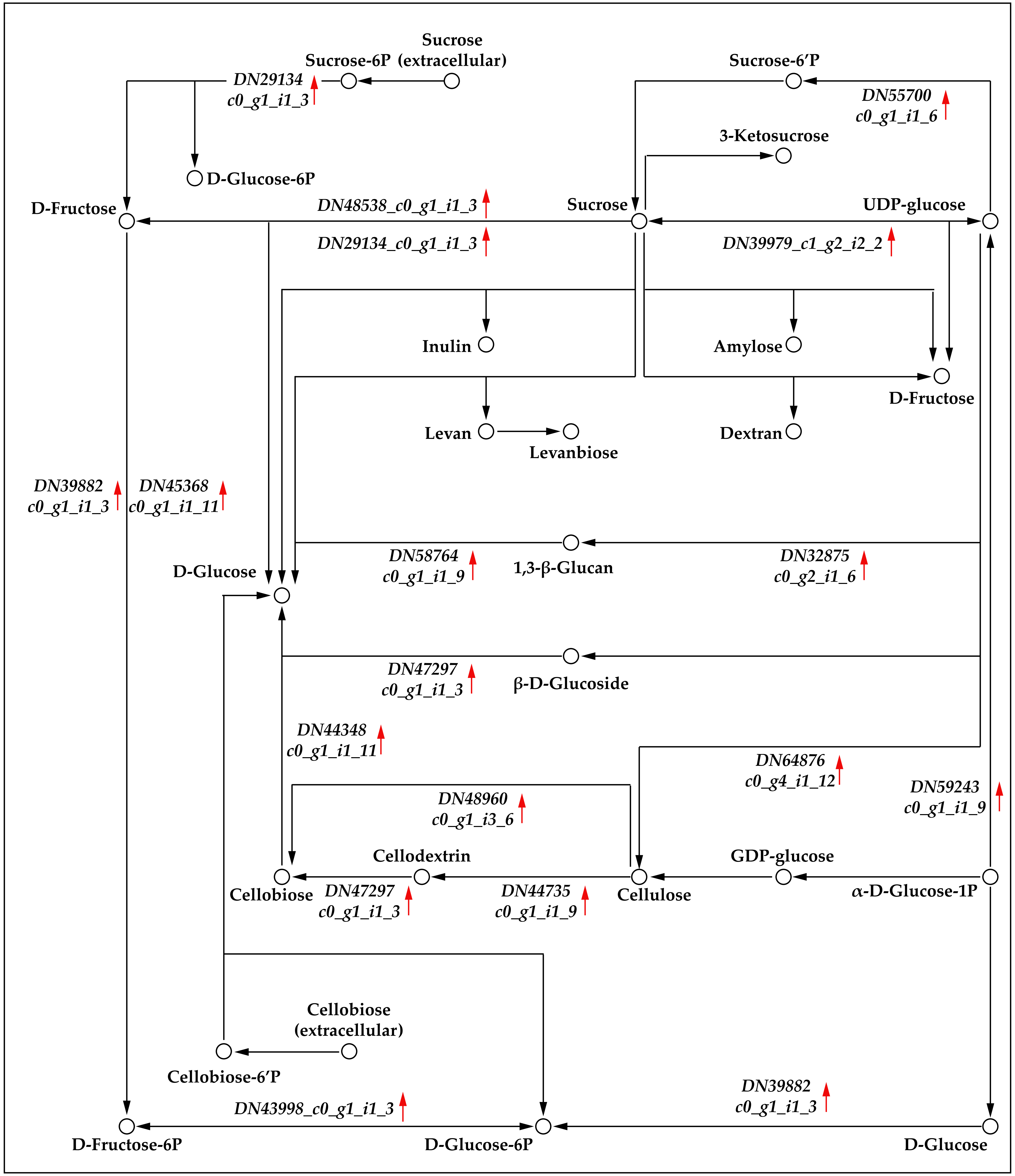

Sucrose (extracellular)
Sucrose-6P
DN29134 c0_g1_i1_3
Sucrose-6′P
DN55700 c0_g1_i1_6
3-Ketosucrose
D-Glucose-6P
D-Fructose
DN48538_c0_g1_i1_3
DN29134_c0_g1_i1_3
Sucrose
UDP-glucose
DN39979_c1_g2_i2_2
Inulin
Amylose
D-Fructose
Levan
Levanbiose
Dextran
DN39882 c0_g1_i1_3
DN45368 c0_g1_i1_11
DN58764 c0_g1_i1_9
1,3-β-Glucan
DN32875 c0_g2_i1_6
D-Glucose
DN47297 c0_g1_i1_3
β-D-Glucoside
DN44348 c0_g1_i1_11
DN64876 c0_g4_i1_12
DN59243 c0_g1_i1_9
DN48960 c0_g1_i3_6
Cellodextrin
GDP-glucose
Cellobiose
DN47297 c0_g1_i1_3
DN44735 c0_g1_i1_9
Cellulose
α-D-Glucose-1P
Cellobiose (extracellular)
Cellobiose-6′P
DN43998_c0_g1_i1_3
DN39882 c0_g1_i1_3
D-Fructose-6P
D-Glucose-6P
D-Glucose
